# Polycomb-mediated repression of EphrinA5 promotes growth and invasion of glioblastoma

**DOI:** 10.1038/s41388-020-1161-3

**Published:** 2020-01-27

**Authors:** Barbara Ricci, Thomas O. Millner, Nicola Pomella, Xinyu Zhang, Loredana Guglielmi, Sara Badodi, Dario Ceric, Carolina Gemma, Erica Cognolato, Ying Zhang, Sebastian Brandner, Michael R. Barnes, Silvia Marino

**Affiliations:** 1 Blizard Institute, Barts and The London School of Medicine and Dentistry, Queen Mary University of London, 4 Newark Street, London, E1 2AT UK; 2 Department of Surgery and Cancer, Imperial College London, Hammersmith Hospital Campus, London, W12 0NN UK; 3 Division of Neuropathology, The National Hospital for Neurology and Neurosurgery, University College London Hospitals NHS Foundation Trust, Queen Square, London, WC1N 3BG UK; 4 Centre for Translational Bioinformatics, William Harvey Research Institute, Barts and The London School of Medicine and Dentistry, Queen Mary University of London, London, EC1M 6BQ UK

**Keywords:** Cancer models, Mechanisms of disease

## Abstract

Glioblastoma (GBM) is the most common and most aggressive intrinsic brain tumour in adults. Integrated transcriptomic and epigenomic analyses of glioblastoma initiating cells (GIC) in a mouse model uncovered a novel epigenetic regulation of EfnA5. In this model, Bmi1 enhances H3K27me3 at the *EfnA5* locus and reinforces repression of selected target genes in a cellular context-dependent fashion. EfnA5 mediates Bmi1-dependent proliferation and invasion in vitro and tumour formation in an allograft model. Importantly, we show that this novel Polycomb feed-forward loop is also active in human GIC and we provide pre-clinical evidence of druggability of the EFNA5 signalling pathway in GBM xenografts overexpressing Bmi1.

## Introduction

Malignant gliomas are the most common intrinsic brain tumours in adults. They grow highly invasively, cannot be completely resected by surgery, and conventional anticancer treatments have limited efficacy, resulting in a dismal overall prognosis. Dysregulation of epigenetic mechanisms, together with genetic mutations, is an essential driver in the progression of malignant gliomas (reviewed in [1]). Therefore, to identify novel druggable targets, it is essential to elucidate the molecular mechanisms of this epigenetic dysregulation.

Polycomb group proteins (PcG) are chromatin associated proteins that maintain hereditable gene repression through histone modification and chromatin remodelling. At least two distinct PcG complexes have been identified, PRC1 and PRC2 (reviewed in [2]). PRC2 is composed of a catalytic subunit, Ezh2, which binds to Suz12 and EED to catalyse trimethylation of histone H3 at lysine 27 (H3K27me3), a bona fide epigenetic silencing mark. PRC1 depends upon PRC2 for recruitment to PcG target genes and is responsible for mono-ubiquitylation of histone H2A at lysine 119 (H2AK119u), an enzymatic activity dependent on the E3 ubiquitin ligase activity of Ring1B, which is enhanced by Bmi1. This sequence of events induces chromatin compaction and inhibition of transcription elongation (reviewed in [3]), although alternative mechanisms of action have also been described [4].

The role of several PcG genes, during the development of the mammalian central nervous system (CNS) and in the maintenance of postnatal stem cells in the adult brain, has been extensively characterised (reviewed in [5]). Loss of function studies in the mouse have shown that Bmi1 is essential for regulation of cell cycle entry of neural progenitors and for self-renewal of neural stem cells (NSC) [6–8]. These actions are, at least in part, mediated through transcriptional repression of the *ink4a* locus, encoding for p16^ink4a^ and p19^arf^ [6], and of the cell cycle inhibitor p21^waf1/cip1^ [9, 10].

Cells with “stem-like” properties have been described in many cancers. These cells are essential for tumour maintenance and they frequently express stem cell genes as well as exhibit a stem cell-like chromatin structure. Bmi1 is highly expressed in glioblastoma stem/initiating cells (GIC) [11] and microRNAs—miR128 and miR218—have been identified, which specifically block glioma self-renewal through Bmi1-downregulation [12, 13]. In keeping with these data, increased tumour latency and a shift towards glioma of a lower histological grade were observed in an experimental murine glial tumour arising in ink4a/arf deficient mice bred into a *Bmi1*^−/−^ background [14]. Interestingly, knockdown of BMI1 in human GIC (hGIC) significantly reduced tumour growth in a xenograft mouse model [15]. The role of Ezh2 in oncogenesis is also well characterised and it has been shown to be multimodal. In gliomagenesis, somatic mutations of histone H3 variant H3F3A have been described in paediatric tumours (DIPGs), leading to depletion of H3K27me3 on canonical H3 because of inhibition of PRC2 activity [16]. EZH2 has also been shown to contribute to the pathogenesis of adult high grade gliomas (HGG) via a non-histone mediated interaction with STAT3. In this case, the trimethylation of STAT3 at K180 by EZH2 was essential for aberrant STAT3 activation in GIC [17], a finding known to be associated with poor survival in patients with GBM [18]. There is likely a complex interplay between BMI1 and EZH2 in GBM and recent evidence shows that strategies that simultaneously target multiple epigenetic regulators may be required to control GBM growth [19].

We have recently demonstrated that conditional overexpression of Bmi1 has a different functional impact on CNS development depending on the differentiation stage of neural precursor cells [20], and that this is mediated by the amount of H3K27me3 at the promoter region of selected target genes in a cell-context-dependent fashion [21]. We have also shown that GIC isolated from a mouse model of HGG [22] show a similar epigenetic regulation of Bmi1 target genes [21]. These data are in keeping with increased H3K27me3 being a general mechanism mediating the functional outcome of elevated Bmi1 expression in both non-neoplastic and neoplastic contexts.

Here we have used a combined genome-wide and target gene-driven approach to comprehensively identify target genes and pathways mediating Bmi1 function specifically in GIC as compared with NSC. The availability of non-neoplastic NSC engineered to overexpress Bmi1 allowed us to mimic the physiological fluctuation of Bmi1 expression during neural differentiation.

## Results

### Differential redistribution of the H3K27me3 mark in mGIC as compared with NSC

In order to define the cellular pathways deregulated in gliomagenesis in a Bmi1-dependent, H3K27me3-mediated manner, we used a well-established mouse model of gliomagenesis that relies on the loss of *PTEN* and *p53*, two of the most common genetic alterations in *IDH* wild-type glioblastoma (GBM) [23]. The model relies on Adeno-Cre-mediated recombination of floxed alleles, either by intraventricular virus injection or by in vitro treatment of NSC prior to their intracerebral injection [22]. HGG develop with good penetrance in this model and cells with tumour initiating properties (mGIC) can be effectively propagated in culture. Our previous findings have shown overexpression of Bmi1 and increased global levels of H3K27me3 in these cells, as compared with NSC [21].

**Fig. 1:**
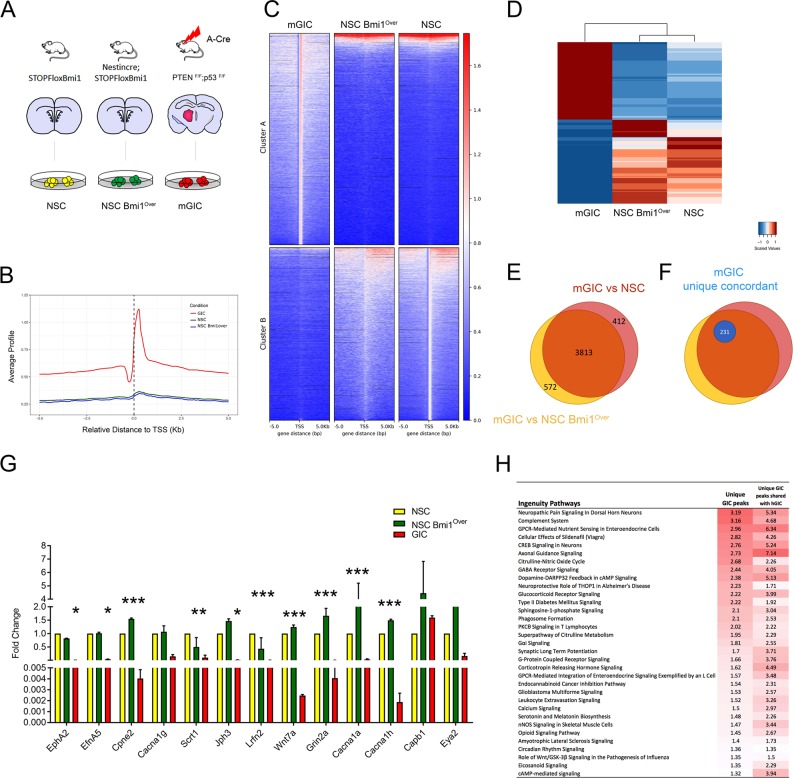
Genome-wide analysis of genes and pathway deregulated in mGIC. **a** Schematic of the experimental setup (non-neoplastic NSC and NSC Bmi1^Over^ as well as neoplastic mGIC). **b** Average profile for H3K27me3 across the three studied genotypes—mGIC, NSC Bmi1^Over^ and NSC—within 5 kb of the TSS. Scores associated with significantly called peaks in mGIC are plotted over the genomic regions of the three genotypes, centred at the TSS. **c** The heatmap for H3K27me3 across the three studied genotypes—mGIC, NSC Bmi1^Over^ and NSC. Scores associated with significantly called peaks in mGIC (top panels, cluster A) and in NSC (bottom panels, cluster B) are plotted over the genomic regions of the three genotypes, centred at the TSS. **d** The heatmap of relative expression of genes deregulated in mGIC across the three studied genotypes—mGIC, NSC Bmi1^Over^ and NSC. **e** Venn diagram showing genes uniquely or commonly repressed in the comparisons mGIC vs NSC Bmi1^Over^ and mGIC vs NSC. **f** Proportion of commonly repressed genes in mGIC concomitantly enriched for H3K27me3 (mGIC unique concordant) is shown in blue. **g** qPCR showing the expression levels of a selection of deregulated genes in biologically independent replicas of NSC, NSC Bmi1^Over^ and mGIC (*n* = 3). *p < 0.05; **p < 0.01; ***p < 0.001, error bars represent ±SEM. **h** IPA analysis identifying canonical pathways specifically and significantly enriched for H3K27me3 in mGIC compared with hGIC of a publicly available dataset (numbers indicate –log(*p* value) and threshold for significance is 1.3; red indicates significantly higher levels of H3K27me3 and white indicates lower).

We performed ChIPSeq for H3K27me3 and RNASeq to investigate the genome-wide correlation between the redistribution of this PRC2 mark and its transcriptional impact in gliomagenesis. To mimic the physiological fluctuation of Bmi1 expression in NSC we compared mGIC to non-neoplastic NSC expressing basal or increased (Bmi1^Over^) levels of Bmi1 (Fig. 1a). Two biologically independent NSC cultures, two NSC Bmi1^Over^ cultures, isolated from *NestinCre;STOPFloxBmi1*, and two mGIC cultures were used for this study.

Analysis of the ChIPSeq datasets using MACS2 identified unique peaks in the neoplastic (cluster A) and non-neoplastic (cluster B) Bmi1 overexpressing context (Fig. 1b, c). Pathway analysis on the ingenuity pathway analysis (IPA) platform identified axonal guidance signalling, glioblastoma multiforme signalling, role of Wnt/GSK-3β signalling and ephrin A signalling, among others, as enriched in mGIC (Fig. S1A).

Comparisons of the transcriptome of mGIC vs NSC and mGIC vs NSC Bmi1^Over^ identified 7319 shared differentially expressed genes (Fig. 1d; 91% and 84% of all deregulated genes, respectively), of which 3813 were specifically downregulated in mGIC (Fig. 1e). We chose to validate 13 genes that were enriched in pathways of interest, or highlighted as likely to be important in GBM pathobiology after thorough literature review. Of these genes, 10/13 were confirmed to be reduced specifically in mGIC in biological replicas of NSC, NSC Bmi1^Over^ and mGIC (Fig. 1g).

To determine the molecular pathways that are transcriptionally regulated by the PRC2-mediated H3K27me3 mark, we integrated the RNASeq and ChIPSeq datasets. This identified a core subset of 231 genes that had acquired the H3K27me3 mark in mGIC but not NSC or NSC Bmi1^Over^ (defined as “unique”), and that also had concomitant reduced expression (defined as “concordant”) (Fig. 1f). These 231 genes showed significant overlap with 33 datasets of the NIH Roadmap Epigenomics H3K27me3 ChIPSeq database (http://www.roadmapepigenomics.org/) (Fig. S1B).

To begin to assess the translational value of our findings in human GBM, this core subset of genes was comparatively analysed in a publicly available H3K27me3 ChIPSeq dataset of hGIC [24]. In total, 97/231 genes shared the mark in both mGIC and hGIC (Fig. S1C), and a high overlap was found in the molecular pathways that were enriched in both mouse and human contexts (Fig. 1h).

### Transcriptional regulation is Bmi1-dependent in a proportion of H3K27me3 marked genes

**Fig. 2:**
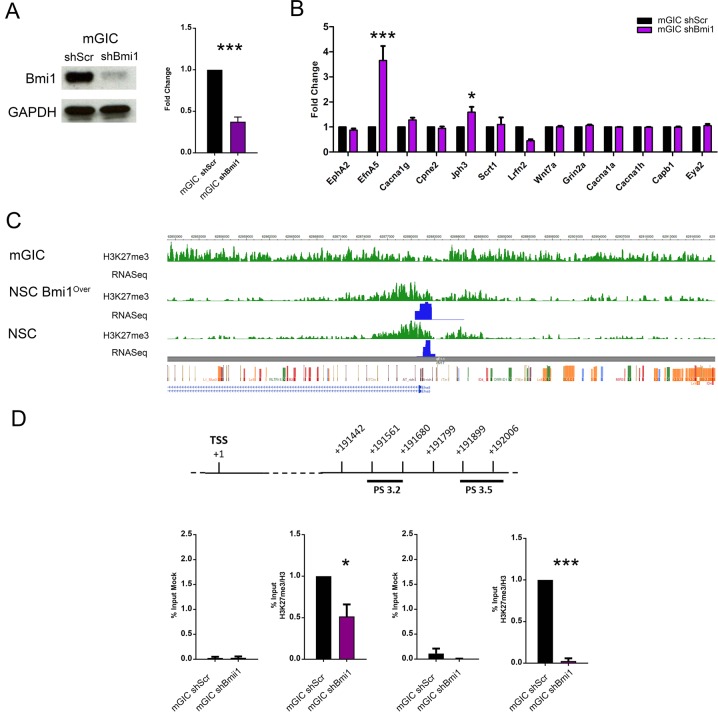
Assessment of Bmi1-dependency of deregulated genes. **a** Western blot (left) and quantification (right) showing effective Bmi1 silencing in shBmi1 mGIC compared with the control shScr (*n* = 4). **b** qPCR analysis of Bmi1-dependency of candidate gene expression (*n* = 3). **c** Visualisation of the EfnA5 locus, centred on the TSS, shows a differential H3K27me3 distribution (green) and expression (blue) between mGIC and the non-neoplastic NSC and NSC Bmi1^Over^. **d** Schematic representation of the EfnA5 region where PS 3.2 and PS 3.5 primer pairs allign (top), and quantification of qChIP assay (bottom) confirms that the H3K27me3 levels depend on Bmi1 expression in mGIC. Immunoprecipitated chromatin (mock) was used as negative control (*n* = 6). *p < 0.05; **p < 0.01; ***p < 0.001; ****p < 0.0001; error bars represent ±SEM.

To assess which of the genes identified in the screening described above were dependent on Bmi1 expression, we silenced Bmi1 in mGIC cultures with shRNA (Fig. 2a) and then assessed the expression levels of the genes previously validated. We found that 2/10 validated genes, EfnA5 and Jph3, were upregulated upon Bmi1 silencing, demonstrating that their regulation is Bmi1-dependent (Fig. 2b). Upregulation of EfnA5 upon Bmi1 silencing was confirmed at the protein level by means of western blot (Fig. S2A).

Our ChIPSeq data showed widespread enrichment for H3K27 trimethylation at the *EfnA5* locus in mGIC, as compared with both NSC and NSC Bmi1^Over^, which was accompanied by reduced expression with RNASeq (Fig. 2c). A similar pattern of H3K27me3 enrichment at the *EFNA5* locus (Fig. S2B) and reduced expression (Fig. S2C) was observed when we analysed published data from hGIC [24].

ChIP for H3K27me3, followed by qPCR for *EfnA5* in mGIC upon silencing of Bmi1, confirmed that H3K27me3 enrichment was dependent on the expression levels of Bmi1 (Fig. 2d).

These data are in keeping with a Bmi1-mediated regulation of *EfnA5* via modulation of the levels of H3K27me3 at its promoter in our GBM mouse model.

### Bmi1 controls H3K27me3 levels at the *EfnA5* locus via downregulation of JmjD3 in mGIC

In order to maintain a cell-type-specific expression pattern, H3K27me3 at specific gene loci is finely regulated by histone methylase and demethylase activity [25, 26]. We have previously demonstrated that NSC overexpressing Bmi1 show reduced expression of the demethylase JmjD3, but no significant changes in expression levels of the methylase Ezh2, suggesting that Bmi1 might control the H3K27me3 repressive mark through the downregulation of JmjD3 [21]. To test the potential contribution of JmjD3 in the regulation of H3K27me3 levels in our model, we performed a proximity ligation assay (PLA) for H3K27me3 on the *EfnA5* locus (Figs. S3 and 3a) upon overexpression of JmjD3 vs treatment with an Ezh2 inhibitor (Ezh2i).

**Fig. 3:**
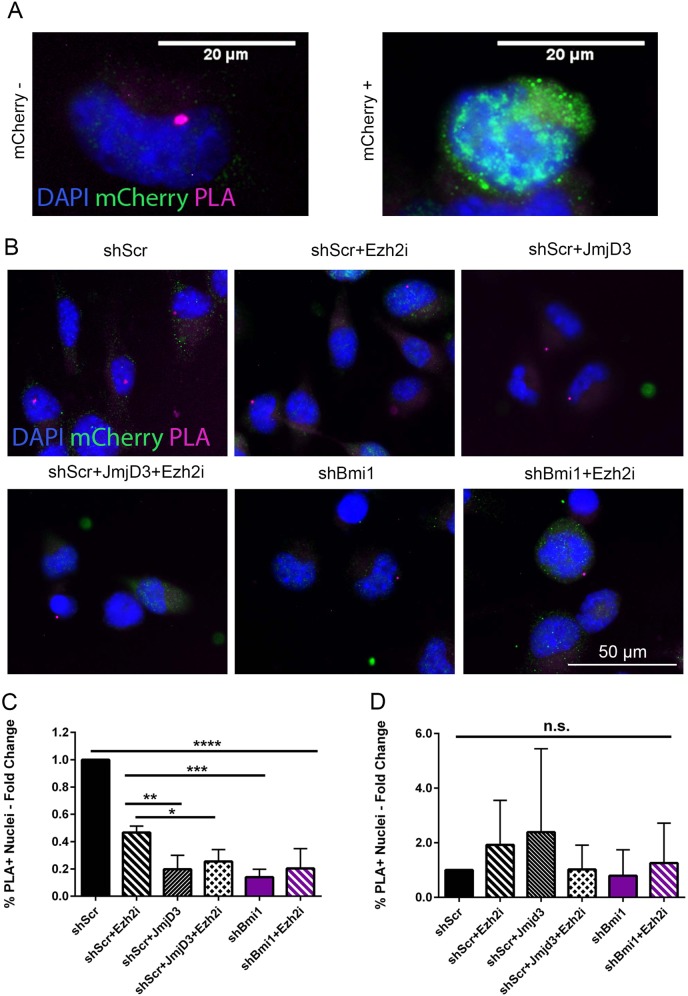
Bmi1 controls the H3K27me3-dependent repression of EfnA5 through downregulation of JmjD3. **a** High resolution images showing shScr cells overexpressing mCherry-JmjD3: the H3K27me3 PLA signal at EfnA5 locus is detectable in mCherry− cell (left panel) and not in mCherry+ cell (right panel). **b** Representative images of the PLA assay in shScr and shBmi1 mGIC in combination with JmjD3 reconstitution and Ezh2 inhibitor treatment (Ezh2i). **c** and **d** Quantification of the percentage of PLA positive nuclei with EfnA5 probe (**c**), or negative probe (**d**), showing the specific modulation of the H3K27me3 levels at *EfnA5* locus (*n* = 6). *p < 0.05; **p < 0.01; ***p < 0.001; ****p < 0.0001; error bars represent ±SEM. Scale bars represent 20 µm in **a** and 50 µm in **c**.

We show that increased expression of JmjD3 in mGIC (shScr + JmjD3; Fig. 3b top right panel and Fig. 3c) led to a significantly reduced H3K27me3 at the *EfnA5* locus as compared with shScr (Fig. 3b top left panel and Fig. 3c) and shScr treated with Ezh2i (shScr + Ezh2i; Fig. 3b top middle panel and Fig. 3c), confirming that JmjD3 plays a major role in regulating the levels of H3K27me3 at the *EfnA5* locus in mGIC. In keeping with this interpretation, cells overexpressing JmjD3 and further treated with Ezh2i (shScr + JmjD3 + Ezh2i; Fig. 3b bottom left panel) did not show any significant additional decrease of the H3K27me3 signal as compared with shScr + JmjD3 only (Fig. 3c). Similar results were observed in both shBmi1 and shBmi1 + Ezh2i cells (Fig. 3b bottom middle and bottom right panels, respectively), showing a decreased level of H3K27me3 upon Bmi1 silencing compared with shScr, but no significant additional change when Ezh2i was added. Off-target effects were also ruled out (Fig. 3d).

We conclude that Bmi1 regulates the levels of H3K27me3 via repression of JmjD3 in mGIC (see Fig. S3B for schematic).

### Bmi1 regulates cell morphology, proliferation and migration/invasion via repression of EfnA5 in mGIC

We have shown above that EfnA5 is upregulated upon Bmi1 silencing. Therefore, we next set out to assess in vitro the functional role of EfnA5 as a mediator of the Bmi1-dependent phenotype of mGIC. We used pre-clustered recombinant mouse EfnA5 Chimera-Fc (rmEfnA5-Fc) to activate EfnA5 forward signalling [27].

**Fig. 4:**
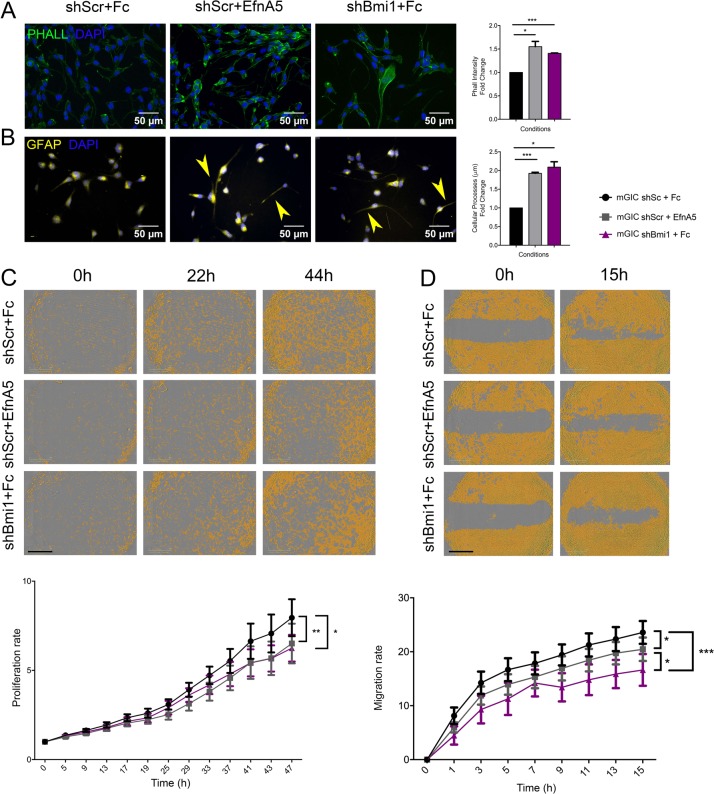
EfnA5 signalling negatively regulates mGIC properties in vitro. **a** Immunocytochemistry for phalloidin (green) and corresponding quantification show that stress fibres increase in shScr upon activation of EfnA5 signalling similarly to shBmi1 (*n* = 3). **b** Immunocytochemistry for GFAP (yellow) and quantification reveal increased cellular processes lengh in mGIC with high levels of EfnA5 (*n* = 3), yellow arrowheads indicate pronounced cellular processes. **c** Representative pictures and quantitative analysis of the proliferation rate upon prolonged treatment with mouse recombinant EfnA5 or Fc control. The yellow mask represents the % of confluence (*n* = 9). **d** Representative pictures and quantitative analysis at different time points after creation of wound and upon prolonged treatment with mouse recombinant EfnA5 or Fc control. Yellow mask represents the % confluence and quantification show the migration rate over time (*n* = 6). *p < 0.05; **p < 0.01; ***p < 0.001; ****p < 0.0001; error bars represent ±SEM. Scale bars represent 50 µm in **a**, **b** and 800 µm in **c**, **d**.

EfnA5 regulates focal adhesion, cell motility and cancer invasion via modulation of the actin cytoskeleton [28, 29], therefore we assessed whether upregulation of EfnA5 upon shBmi1 affected cellular architecture in our model. We evaluated the formation of stress fibres, with phalloidin staining, as a measure of EfnA5-mediated activation of focal adhesion as well as cellular process length with GFAP staining. When we compared rmEfnA5-Fc treated mGIC with shBmi1 mGIC, phalloidin intensity was similar (Fig. 4a), and cellular process length was increased in both conditions. These data support the interpretation that exogenous EfnA5 modifies the cytoskeleton and cell morphology in a similar fashion to Bmi1 knockdown, which leads to an endogenous overexpression of EfnA5.

Next, we assessed whether EfnA5 repression contributed to the previously described [15] Bmi1-mediated regulation of cellular proliferation. mGIC treated with rmEfnA5-Fc showed significantly decreased proliferation, with a similar decrease observed in shBmi1 mGIC (Fig. 4c). Expression of p16^ink4a^, p19^arf^ and p21 was not significantly upregulated in this model (Fig. S2D).

In addition, shBmi1 mGIC showed reduced migration potential (Fig. 4d), in keeping with previous reports [14]. A similar phenotype was also observed when mGIC were treated with rmEfnA5-Fc, raising the possibility that modulation of EfnA5 expression levels may contribute to Bmi1-mediated regulation of cell migration.

**Fig. 5:**
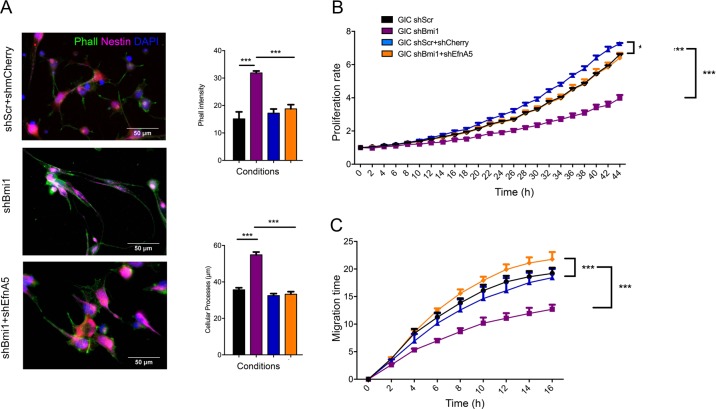
EfnA5 silencing rescues Bmi1kd mGIC properties. **a** Immunocytochemistry and corresponding quantification for phalloidin (green) and Nestin (magenta) shows that the increase of stress fibres and cellular processes length depends on the upregulation of EfnA5. Endogenous mCherry is shown in red (*n* = 3). **b** Quantitative analysis of the proliferation rate showing that Bmi1 promotes mGIC proliferation by repressing EfnA5. (*n* = 6). **c** Quantification of the migration rate reveals that high EfnA5 expression inhibits the migratory capacity of mGIC (*n* = 6). *p < 0.05; **p < 0.01; ***p < 0.001; ****p < 0.0001; error bars represent ±SEM. Scale bars represent 50 µm in **a** and 100 µm in **b**.

To assess whether the upregulation of EfnA5 expression observed upon Bmi1 silencing was responsible for the observed phenotype, we used shRNA to knockdown EfnA5 in shBmi1 mGIC (Fig. S4A; “shEfnA5”, with “shmCherry” as the reporter-matched scrambled control). Interestingly, silencing of EfnA5 in shBmi1 mGIC, neutralised the effect of Bmi1 knockdown on stress fibre formation and cellular process length (Fig. 5a). Importantly, the decreased proliferation rate observed in shBmi1 mGIC, as assessed by live imaging (Figs. 5b and S4B) and neurosphere assay (Figs. S4D and S5A), was also rescued when concomitant silencing of EfnA5 was carried out, a finding supported by the lack of significant impact of Bmi1 silencing on the expression of cell cycle inhibitors in our model (Fig. S2D). Silencing of EfnA5 in shBmi1 mGIC also rescued the migration defect observed in shBmi1 mGIC (Figs. 5c and S4C). Finally, shBmi1 mGIC showed reduced invasion through a 3D collagen gel, which was also rescued by concommitant shEfnA5 (Fig. S5B).

Taken together our data show that repression of EfnA5 plays a key role in mediating Bmi1 function in the regulation of key mGIC properties in vitro.

### Repression of EfnA5 by Bmi1 is essential for glioblastoma development in an allograft model

**Fig. 6:**
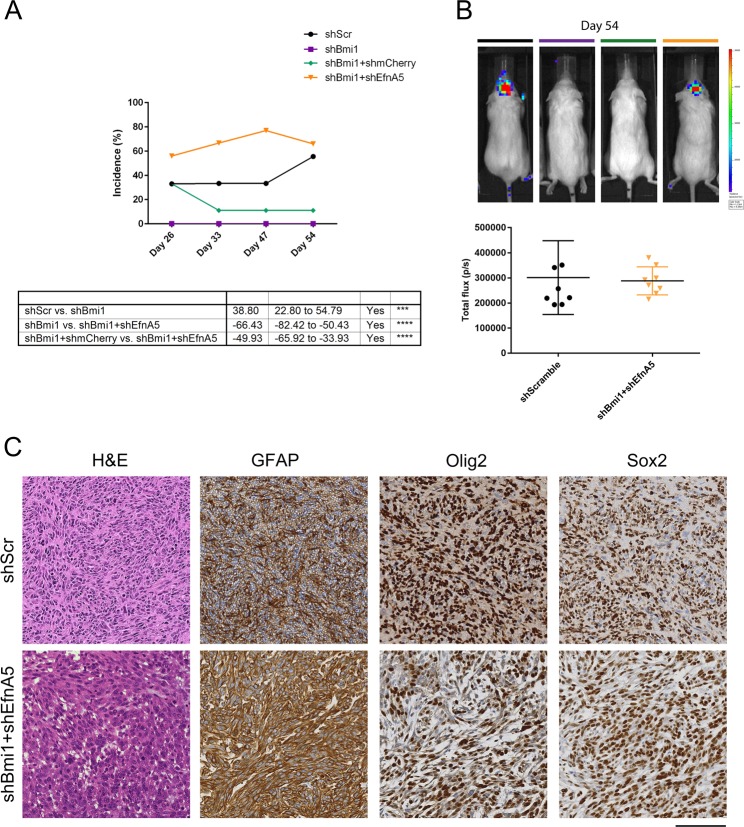
EfnA5 repression mediates Bmi1 modulated tumour development in an allograft model. **a** Tumour incidence was monitored over a representative number of time points with BLI (shScr and shBmi1 *n* = 9; shBmi1 + shmCherry and shBmi1 + shEfnA5 *n* = 8). **b** Representative BLI images of NODSCID mice 54 days post-injection of mGIC with different expression levels of EfnA5 and corresponding (**b**) frequency distribution plot (shScr and shBmi1 *n* = 9; shBmi1 + shmCherry and shBmi1 + shEfnA5 *n* = 8). **c** Histology of representative tumour areas, H&E shows pleomorphic glial cells with compacted tumour growth with necrosis; IHC shows tumour cells are strongly positive for GFAP, Olig2 and Sox2, confirming their glial origin. Scale bar = 250 µm.

Given these important roles in proliferation, migration and invasion, we asked whether EfnA5 might also mediate the impact of Bmi1 on tumourigenesis in vivo. Analytical imaging of NODSCID mice with orthotopically implanted luciferase-tagged mGIC revealed that while 5/9 mice injected with control mGIC developed tumours and silencing of Bmi1 strongly suppressed tumour growth (0/9), concomitant silencing of EfnA5 and Bmi1 rescued tumour incidence (6/9) (Fig. 6a, b). Histological analysis of the engrafted brains revealed high grade glial tumours composed of cells with enlarged, pleomorphic and occasionally hyperchromatic nuclei, frequent mitoses and areas of necrosis (Figs. 6c and S6A, B). Immunostaining for GFAP, Olig2 and Sox2 confirmed the glial nature of these neoplasms (Fig. 6c).

These data show that repression of EfnA5 plays a key role in mediating the tumourigenic role of Bmi1 in vivo.

### BMI1 also regulates cell proliferation via repression of EFNA5 in hGIC

**Fig. 7:**
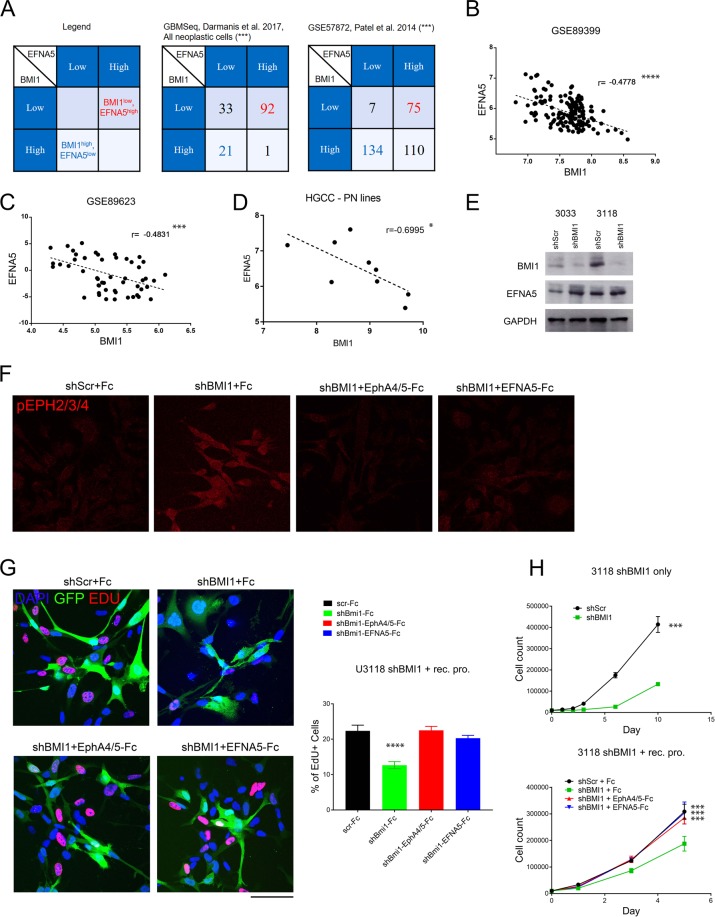
EFNA5 mediates BMI1 function in hGIC. **a** Contingency tables for the expression levels of BMI1 and EFNA5 in GBM cells from single-cell RNAseq datasets (example table top left); levels of significance of negative association using both the Fisher’s exact test and Barnard’s test are in parenthesis. Scatter plots with linear regression statistics showing the correlation between levels of BMI1 and EFNA5 for two hGIC datasets GSE89399 (**b**) and GSE89623 (**c**), and for microarray data from hGIC of the Proneural subgroup from the HGCC (**d**). **e** Levels of BMI1 and EFNA5 by western blot for the two hGIC lines, U3033 and U3118, after BMI1 knockdown, GAPDH was used as a control. **f** Immunofluorescence staining in U3118 cells for phosphorylated EphA receptors 2, 3 and 4 is shown after BMI1 knockdown and EFNA5 pathway inhibition with recombinant protein (*n* = 3). **g** Representative images and quantification for EdU staining in U3118 cells after BMI1 knockdown and EFNA5 pathway inhibition, showing an increase in downstream pathway activation in the condition with highest EFNA5 expression and no EFNA5 pathway blockade (*n* = 3). **h** Proliferation assays for U3118 after BMI1 knockdown with shRNA (upper) and concomitant BMI1 knockdown and EFNA5 pathway inhibition with recombinant proteins (lower) (*n* = 3). *p < 0.05; **p < 0.01; ***p < 0.001; ****p < 0.0001; error bars represent ±SEM. Scale bar is 250 µm in **f**, **g**.

To understand whether our findings could be translatable to human GBM, we assessed whether a significant correlation existed between BMI1 and EFNA5 expression in human tumours. We used published RNA microarray, RNASeq and single-cell RNASeq datasets from The Cancer Genome Atlas (TCGA), the National Center for Biotechnology Information Gene Expression Omnibus databanks and gbmseq.org [30]. Of the TCGA datasets, we found that the dataset with the highest number of tumour samples (hthgu133a, *n* = 548) showed a strong and significant inverse correlation between BMI1 and EFNA5 (Fig. S7A), whilst the other three datasets did not (data not shown). Next, we interrogated two independent GBM single-cell RNAseq datasets [30, 31], and confirmed that a significant number of tumour cells displayed a negative association between BMI1 and EFNA5 (Figs. 7a and S7B). Furthermore, we sought to establish whether this negative correlation was found in hGIC specifically by analysing two datasets of primary patient-derived cultured hGIC characterised with RNA microarray (GSE89399 [32]) and RNASeq (GSE89623 [33]). Both of these independent hGIC cohorts displayed a strong and significant negative correlation between BMI1 and EFNA5 (Fig. 7b, c).

Next, we tested whether the functional role for the Bmi1/EfnA5 axis observed in mGIC was also present in hGIC in vitro. To this end, the Human Glioblastoma Cell Culture (HGCC.se [34]) resource was interrogated. An inverse correlation between the expression of BMI1 and EFNA5 was again observed, although it did not reach statistical significance (data not shown). Interestingly, these 48 hGIC lines could be clustered into two subgroups on the basis of the expression levels of BMI1 and EFNA5, with a strong and significant negative correlation of BMI1 and EFNA5 being observed in primary lines belonging to the Proneural molecular subtype (*n* = 9; Fig. 7d).

We selected two of these primary Proneural hGIC lines (U3118 and U3033) to examine the translational potential of the BMI1/EFNA5 pathway. Upon BMI1 knockdown with shRNA, we observed a corresponding increase in EFNA5 levels (Fig. 7e), and a significant decrease in proliferation in both lines (Figs. 7h and S7D). Ephrin downstream signal transduction is activated by a complex process that requires the assembly of higher-order ligand and receptor clusters for signalling initiation [27]. Therefore, pre-clustering of recombinant EfnA5 protein (as we have used with mGIC above) results in activation of the signalling pathway. However, if recombinant protein is added without prior clustering it blocks the ephrin receptor sites and inhibits ephrin pathway signalling [35]. In both lines, activation of the EFNA5 signalling pathway was observed upon BMI1 knockdown, which was rescued by pathway blockade with recombinant proteins, as confirmed by immunofluorescence staining for phosphorylation of selected Eph receptors (U3118 shown in Fig. 7f). We used recombinant chimera-Fc protein for human EFNA5 as well as two of its receptors, EPHA4 and EPHA5, for this pathway inhibition. We show that the decreased proliferation rate observed in hGIC after BMI1 knockdown was rescued with concomitant inhibition of the EFNA5 signalling pathway in U3118 and U3033 (Figs. 7h and S7D, respectively). These data were supported by EdU staining that showed the same decrease upon BMI1 knockdown and rescue with EFNA5 pathway inhibition (Figs. 7g and S7C).

These data are in keeping with the interpretation that repression of EFNA5 also plays an important role in mediating BMI1 function in hGIC.

### Doxazosin effectively targets BMI1^high^/EFNA5^low^ hGIC in vitro and in vivo

**Fig. 8:**
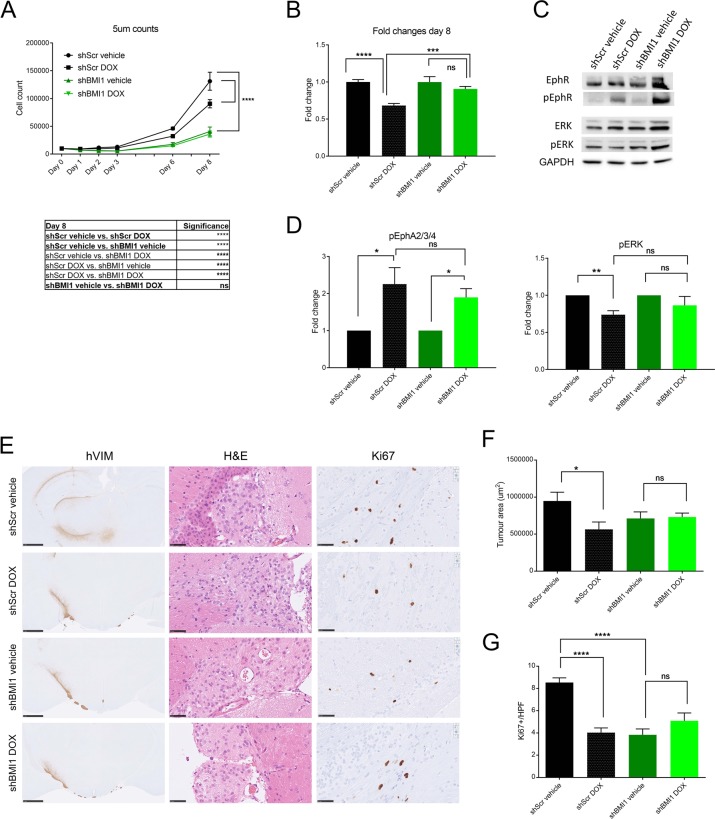
Doxazosin effectively targets BMI1^high^/EFNA5^low^ hGIC in vitro and in vivo. **a** U3118shScr (black) and U3118shBMI1 (green) cells were both treated with either vehicle control or doxazosin (DOX) 5 µM and counted at selected time points (*n* = 3). **b** At day 8, the number of U3118shScr DOX treated cells, as a fraction of U3118shScr vehicle treated cells, was significantly less than the number of U3118shBMI1 DOX treated cells as a fraction of U3118shBMI1 vehicle treated cells (*n* = 3). Western blot (**c**) and quantitative analysis (**d**) showing the levels of EphA2–4, phosphorylated EphA2–4, ERK1/2 and phosphorylated ERK1/2 for the same conditions as in (A) (*n* = 3). **e** Histology of representative tumour areas with human Vimentin immunohistochemistry, H&E and Ki67; scale bars are 1 mm for hVIM and 50 µm for H&E and Ki67 (*n* = 8). **f** Tumour volume assessed by automated quantification of human vimentin immunohistochemistry. **g** Tumour proliferation assessed by Ki67 count per high power field (HPF). *p < 0.05; **p < 0.01; ***p < 0.001; ****p < 0.0001; error bars represent SEM.

Next, we set out to test the hypothesis that pharmacologically targeting the EFNA5 pathway is effective against hGIC expressing high BMI1 levels. In cells with high levels of BMI1, EFNA5 is repressed, therefore we identified a drug that mimicked EFNA5 action. Doxazosin is a small molecule agonist for Eph receptors for which EFNA5 acts as a ligand (EphA2 and EphA4) [36], independent of the α1-adrenoceptor action for which it is commonly used clinically. Upon treatment of U3118shScr and U3118shBMI1 cells with 5 µM doxazosin, proliferation was significantly more impacted in the U3118shScr than it was in the shBMI1 condition (Fig. 8a). Indeed, there was a significant large decrease in proliferation with doxazosin treatment in the shScr group, whilst there was only a small, non-significant difference in the shBMI1 group after eight days (Fig. 8b). Assessment of EphA2–4 phosphorylation (targets of EFNA5) in shScr and shBMI1 cells upon exposure to doxazosin confirmed activation of the pathway (Fig. 8c, d). Decreased levels of phosphorylation of ERK1/2, a known downstream effectors of EphA2 [36], were also detected in shScr cells that were treated with doxazosin, whilst in shBMI1 cells this was not the case (Fig. 8c, d).

We next wanted to see if the results we had observed with doxazosin in vitro could be translated to an in vivo setting. Firstly, we established that doxazosin could cross the blood–brain barrier (BBB) in mice by assessing the levels of the drug in the serum and brain homogenate by liquid chromatography–mass spectrometry after subcutaneous injections of doxazosin (50 mg/kg). The levels of doxazosin in the serum of NODSCID mice reached a peak of nearly 4000 ng/mL, 1 h after injection, whilst the levels in brain homogenate reached a peak of ~50 ng/mg after 2 h (Fig. S8A), a timeframe comparable to that seen in rats [37]. Only approximately 1% of the doxazosin in the serum was entering the brain after subcutaneous injection of doxazosin. The BBB efflux transporter inhibitor elacridar has been shown to be effective pre-clinically in enhancing brain accumulation upon dual administration with several anti-glioma agents [38]. Although it is not known if doxazosin is excreted by these transporters, prazosin, another commonly used α1-adrenoreceptor antagonist sharing high structural similarity with doxazosin, is known to be excreted by these transporters [39, 40]. Two doses of doxazosin, 50 and 100 mg/kg, were combined with either a vehicle control or 100 mg/kg of elacridar (given by oral gavage 4 h before doxazosin dose), and the levels of doxazosin were again measured in the serum and brain homogenate of mice. The levels of doxazosin in the serum were not significantly different across the groups, whilst the levels of doxazosin were significantly greater in brain homogenate when doxazosin was combined with elacridar (Fig. S8B), reaching levels nearly 10 times higher than doxazosin alone, at the higher doxazosin dose. We also found that, in agreement with other studies [36, 41], doxazosin did not have any significant side effects in experimental animals.

We intracranially injected 0.5 × 10^6^ U3118shScr and U3118shBMI1 cells into NODSCID mice. U3118 xenografts have a median survival of ~37 weeks [32]. To assess the effect of doxazosin at an early tumour stage, at 16 weeks eight mice of each shScr and shBMI1 were assigned to either 100 mg/kg elacridar + vehicle, or 100 mg/kg elacridar +100 mg/kg doxazosin. Mice were culled after treatment and tumour volume (immunohistochemistry for human vimentin) and proliferation (Ki67 staining) and apoptosis (cleaved caspase3 staining) were assessed. Tumour volume was significantly reduced after doxazosin treatment in the shScr cells, whereas there was no significant reduction in shBMI1 xenografts (Fig. 8e, f). Proliferation was reduced upon drug treatment in shScr group but not in the shBMI1 group (Fig. 8e, g). No evidence of cCASP3 positive cells was found. All tumours showed cells with enlarged, pleomorphic and occasional hyperchromatic nuclei on H&E, whilst all conditions show diffuse staining for GFAP (cytoplasmic) and SOX2 (nuclear), with a smaller fraction positive for OLIG2 (Fig. S8C).

These data provide pre-clinical evidence in GBM xenografts that the EFNA5 agonist, doxazosin, is effective against early stages of GBM derived from GIC with a BMI1^high^/EFNA5^low^ molecular signature.

## Discussion

We show here a novel epigenetic regulation of EfnA5 in a mouse model of GBM. This highlights a novel Polycomb feed-forward loop in gliomagenesis, whereby PRC1 reinforces repression of selected target genes in a cellular context-dependent fashion.

Consistent with the critical roles of P53 and PTEN-PI3K-AKT alterations in GBM pathogenesis, GBM genomic and proteomic profiles from TCGA show significant correlation between higher levels of AKT activation and worse prognosis in patients with *P53* mutations [42]. To model these pathway alterations, mGIC isolated from HGG arising from NSC/NPC upon intraventricular Adeno-Cre mediated recombination of *p53*^F/F^/*PTEN*^F/F^ [22] were used. We demonstrate that repression of EfnA5 expression via increased trimethylation of H3K27 is a core mechanism mediating the functional outcome of the high levels of Bmi1 seen in *p53*^−/−^;*PTEN*^−/−^ mGIC. We did not observe an impact on ink4a/arf in this model, in keeping with existing literature showing a non-functional ink4a/arf pathway in a *p53* knockout setting [16, 43].

The ephrin/Eph receptor family comprises eight ligands (Efn: Eph receptor interacting ligand) and 14 type I transmembrane receptor tyrosine kinases (Eph: erythropoietin-producing human hepatocellular receptors), which are classified into type A and B, whereby nine EphA and five EphB receptors promiscuously bind five EfnA ligands and three EfnB ligands, respectively (reviewed in [44]). Ephrins and Eph receptors are variably expressed in different cells at varying stages of differentiation and play essential roles in the control of cell morphology, adhesion, movement, proliferation and differentiation in embryonic development and tissue homoeostasis. In addition, they are often upregulated in injured tissues, where they inhibit regenerative processes and promote angiogenesis. They are known to be frequently deregulated in cancer, including GBM, being either overexpressed or downregulated (reviewed in [45]). Moreover EphA2 and EphA3, receptors for the ligand EFNA5, are markers of worse outcome in GBM and are involved in proliferation, invasion and neovascularisation [46]. The regulatory mechanisms governing the expression of ephrins and Eph receptors have been extensively studied. The transcription factors HOXA1 and HOXB1 have been shown to activate EphA2 expression in the developing mouse brain [47]. Hypermethylation at CpG islands of promoter regions of many ephrins, including EfnA5, and Eph receptors have been demonstrated in acute lymphoblastic leukaemia [48] and miRNAs, such as miR-210 and miR-26b, have been shown to downregulate the expression of EfnA1 in hepatic ischaemia [49] and EphA2 in gliomas [50]. Importantly, Bmi1 is known to regulate miRNAs, such as miR10a [51] that has been shown to repress EphA8 in glioma cells, mediating the epithelial–mesenchymal transition and promoting cell migration and invasion [52]. *EfnA5* is reported as a miR-10a-3p target in the miRBase database (http://www.mirbase.org/).

Using a genome-wide approach, we show that a novel regulation of *EfnA5* via PcG-mediated histone tail modification is specific to the neoplastic context, and that normal NSC and NSC overexpressing Bmi1 did not reveal similar regulation (Fig. S3B). We also found that the repression of EfnA5 by Bmi1 contributes to tumour growth in vitro and in vivo in our mouse model.

The aberrant retention of H3K27me3 as a common epigenetic mechanism mediating the phenotype of Bmi1 overexpression opens a novel conceptual entry point into the interrogation of the role of Bmi1 in gliomagenesis. We show that repression of JmjD3 significantly contributes to the increased H3K27me3 at selected target genes in a mouse model. JmjD3 is the first demethylase to have been shown to antagonise Polycomb silencing, and is required for early neural commitment [53, 54]. ChIPSeq experiments have shown that it is a direct Bmi1/PRC1 target in various model systems [55, 56]. JmjD3 also plays a critical role in late neurogenesis [57]. JmjD3 is induced during differentiation of hGIC, where it promotes a differentiation-like phenotype via chromatin dependent (*ink4a/arf* locus activation) and chromatin independent (nuclear p53 protein stabilisation) mechanisms [58]. Moreover, ChIPSeq analysis in embryonic stem cells expressing a GFP-tagged version of JmjD3, revealed that JmjD3 targets are enriched in molecular pathways that are critically involved in gliomagenesis. These include the direct regulation of *Mdm2* and *Akt2* as well as the regulation of genes such as *Stat3* and *Rhpn2*, which are involved in GBM Mesenchymal transformation and as downstream effectors of GBM genomic lesions, respectively [59, 60]. Finally, pharmacological inhibition of JmjD3 has been shown to impair the tumourigenic potential of H3K27M DIPG cells by restoring normal levels of H3K27me3 [61].

Our data delineate a novel PcG feed-forward loop in which Bmi1 enhances its repressive efficacy at specific target genes in mGIC by increasing H3K27me3-mediated gene repression. We show that the modulation of EfnA5 expression via regulation of H3K27me3 levels is mGIC specific, thus raising the possibility that a multi-layered regulatory mechanism is at play at a locus playing a key role in gliomagenesis.

We also report that an inverse correlation of gene expression of BMI1/EFNA5 is found in human GBM and in hGIC, and we provide initial evidence that repression of EFNA5 mediates, at least in part, the role of BMI1 in regulating hGIC proliferation. Existing literature in human cells has shown that EFNA5 acts as a tumour suppressor gene in GBM via negative regulation of EGFR [62], thus providing an interpretative framework for the requirement of an epigenetic regulation of this protein in GBM.

The wealth of suggestive evidence linking perturbations in H3K27 methylation to the development of malignant gliomas indicates that it will be essential to further explore whether and how this epigenetic mark could be a rational target for epigenetic therapy to counteract tumour maintenance in GBM. Moreover, Eph receptor/ephrin signalling pathways are a promising area for anticancer therapies with strategies for their therapeutic targeting already developed with some already in clinical trials [63]. Here we show that doxazosin, an EFNA5 mimic, inhibits proliferation of GIC in a BMI1-dependent fashion. We demonstrate that doxazosin inhibits the ERK pathway, as assessed by its decreased phosphorylation. We also show that doxazosin is able to cross the BBB in combination with an efflux transporter inhibitor. When GIC were orthotopically xenografted into NODSCID mice, treatment with doxazosin recapitulated the in vitro findings in early stage tumours: tumour size was significantly decreased in the shScr tumours with concomitant decreased proliferation, whilst there was no difference in shBMI1 tumours. Together these results provide pre-clinical evidence that precision targeting of Eph receptor/ephrin signalling [64] could be an effective therapeutic tool in GBM overexpressing BMI1.

## Material and methods

### Generation of mice and genotyping

Transgenic *STOPFloxBmi1* mice were previously generated in our laboratory [21]. Activation of Bmi1 overexpression was obtained in embryos by crossing *STOPFloxBmi1* and *NestinCre* mice to generate double transgenic animals, as previously described [21].

### Cultures

Primary NSC cultures were prepared from E16.5 *STOPFloxBmi1;NestinCre* transgenic and control wild-type embryos. Primary mouse *PTEN*^*F/F*^;*P53*^*F/F*^ NSC and mGIC were cultured as adherent cells in Neurobasal and DMEM/F12 media containing N2 and B27 supplements and human recombinant FGF and EGF.

### ChIPSeq and RNASeq

RNASeq: after trimming and quality control, reads were aligned to the mm10 mouse genome using STAR. Trimmed mean of *M*-values normalisation was applied to the dataset and differential expression analysis was performed using the Bioconductor package edgeR in R [65], with a quasi-likelihood *F*-test and an FDR cut-off of 0.05. GEO Record number: GSE141961.

ChIPSeq: after trimming and quality control, reads were aligned to the mm10 mouse genome using Bowtie v2.3.4 (sourceforge.net/projects/bowtie-bio/files/bowtie2/), allowing up to one mismatch per read and discarding multi-mapped reads. The MACS2 algorithm [66] was used to call H3K27me3 peaks (subroutine *callpeak*) and perform the differential binding analysis (subroutine *bdgdiff*). GEO Record number: GSE141961.

### Proximity ligation assay

We cloned a 578 bp region spanning chr17:62687621-62688198 for EfnA5 locus into a TOPO TA Cloning vector. One microgram of plasmid DNA was used as template to generate a biotintylated probe. Slides with attached cells were then incubated with probes, followed by ligation and amplification steps. Anti-mCherry antibody was used to detect transfected cells.

### In vitro functional assays

Proliferation, wound healing scratch assay and invasion assay for mGIC were imaged with IncuCyte ZOOM/Live-Cell Software (EssenBioScience) or INCell 2200 (GE Healthcare) with Developer Toolbox software (GE Healthcare).

### Orthotopic transplantation of GIC into NODSCID mice and bioluminescence imaging (BLI)

Six- to twelve-week-old NODSCID mice were anaesthetised and 5 × 10^5^ mGIC were injected into the right cerebral hemisphere with the following coordinates from the bregma suture: 2 mm posterior, 2 mm lateral, 4 mm deep, 10° angle. Tumour formation and growth was assessed by BLI for mGIC xenografts.

### In vivo treatment with doxazosin

100 mg/kg of doxazosin was given daily by subcutaneous injection and 100 mg/kg elacridar was given by oral gavage every second or third day, 4 h prior to doxazosin dose. Experimental animals (*n* = 8 for each group) were treated with elacridar and vehicle control, or elacridar and doxazosin. Mice were culled after treatment and brains removed for histological assessment.

All further methods and additional details are included as supplementary material.

## Supplementary information

S1

S2

S3

S4

S5

S6

S7

S8

Supp Figure legends

M&M
