# Supplementary figures and images for: Polycomb-mediated repression of EphrinA5 promotes growth and invasion of glioblastoma
Source: Oncogene. 2020 Jan 27;39(12):2523–38. doi: 10.1038/s41388-020-1161-3 (PMC7082224; doi:10.1038/s41388-020-1161-3)

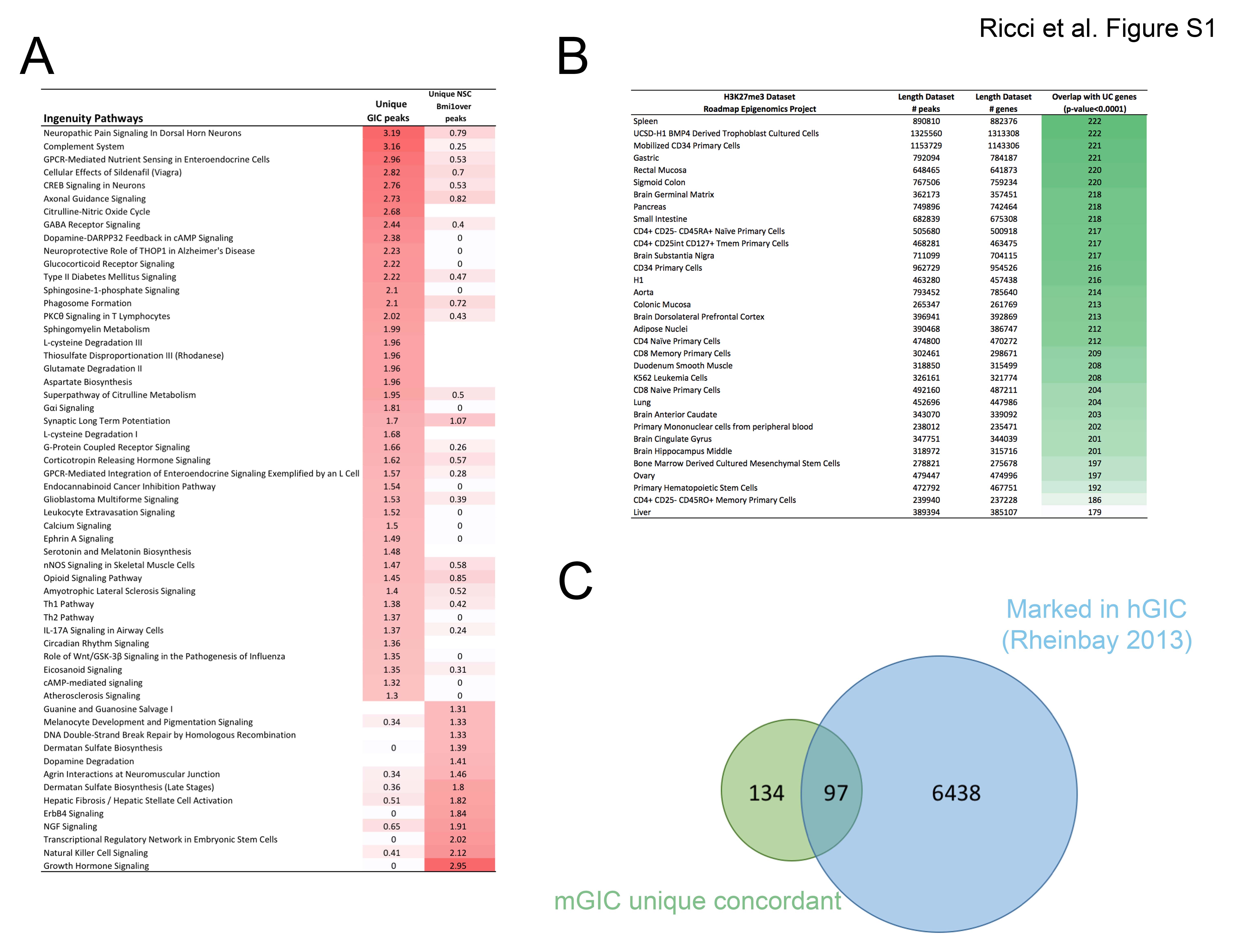

Supplement: Supplementary file 1 — S1 [file 41388_2020_1161_MOESM1_ESM.jpg]

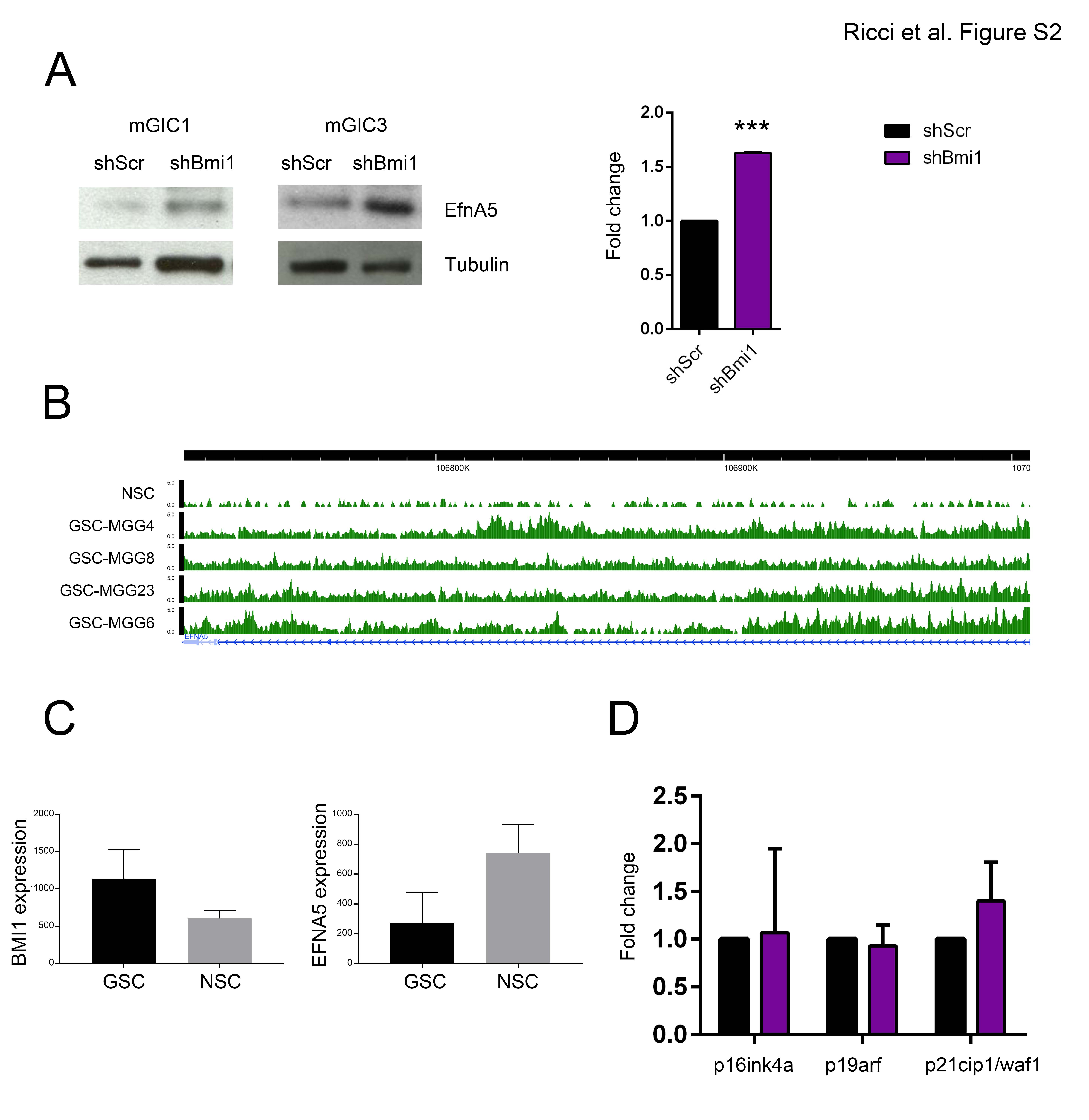

Supplement: Supplementary file 2 — S2 [file 41388_2020_1161_MOESM2_ESM.jpg]

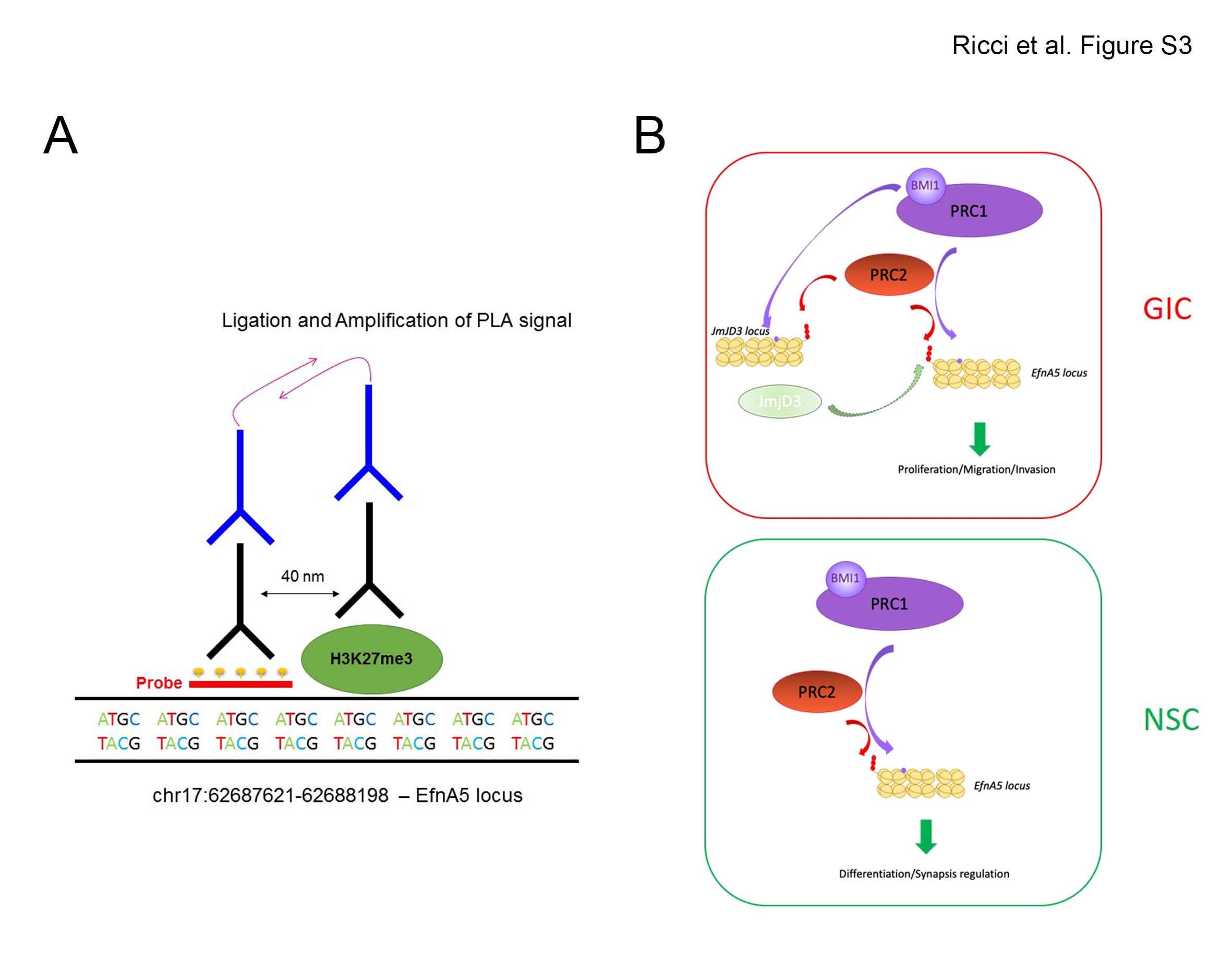

Supplement: Supplementary file 3 — S3 [file 41388_2020_1161_MOESM3_ESM.jpg]

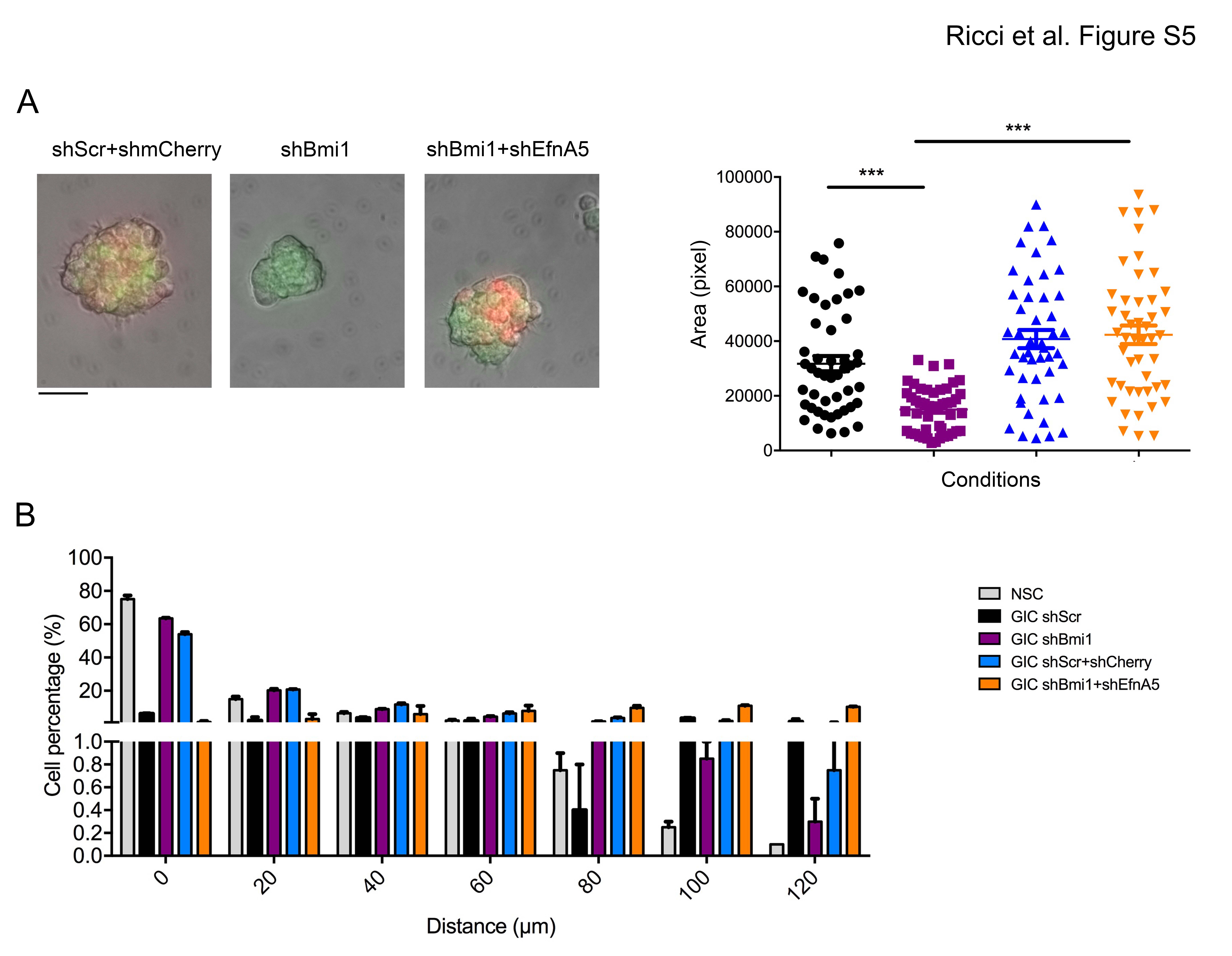

Supplement: Supplementary file 5 — S5 [file 41388_2020_1161_MOESM5_ESM.jpg]

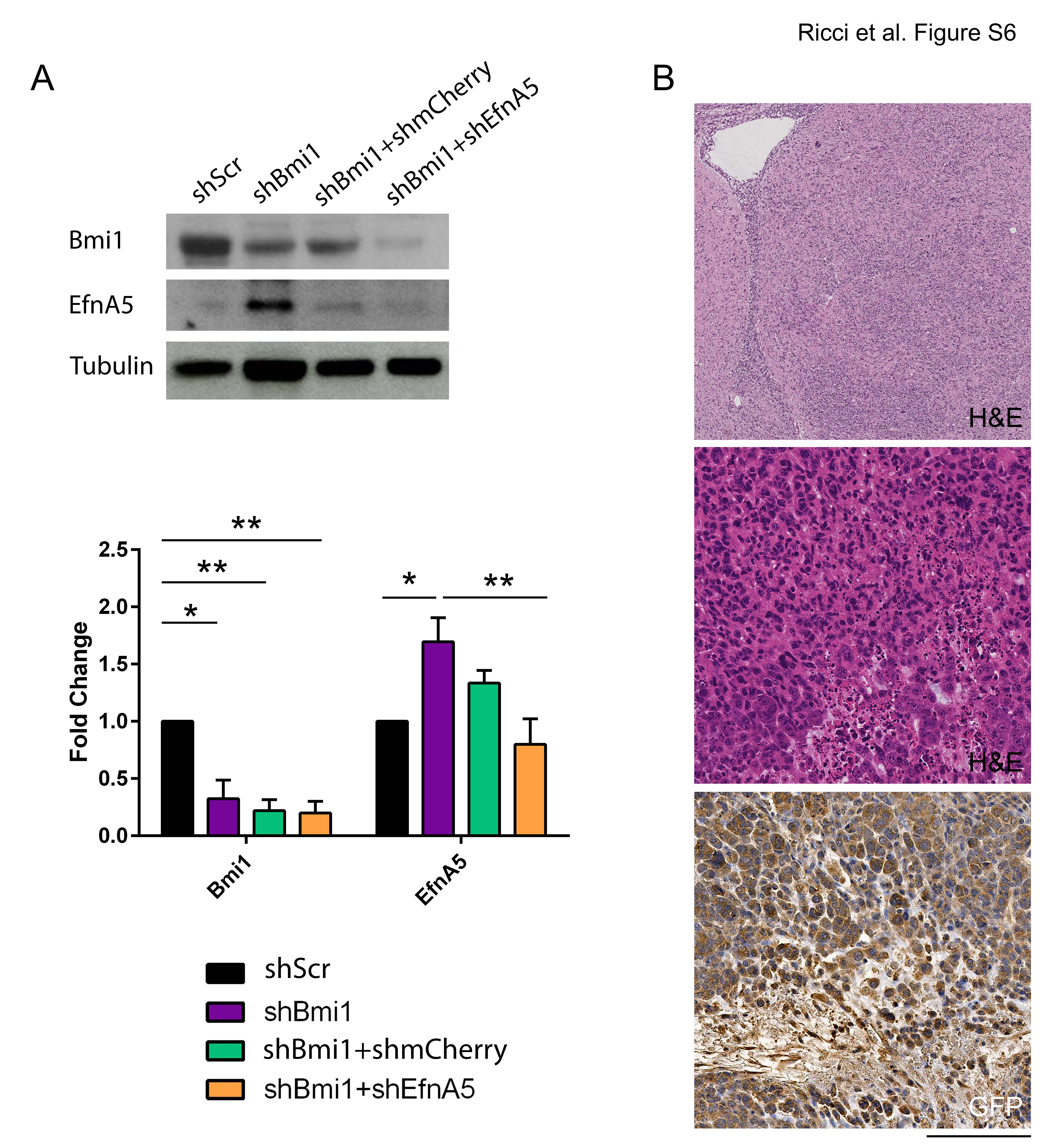

Supplement: Supplementary file 6 — S6 [file 41388_2020_1161_MOESM6_ESM.jpg]
