# Supplementary material for: Polycomb-mediated repression of EphrinA5 promotes growth and invasion of glioblastoma
Source: Oncogene. 2020 Jan 27;39(12):2523–38. doi: 10.1038/s41388-020-1161-3 (PMC7082224; doi:10.1038/s41388-020-1161-3)
Supplement: Supplementary file 9 — Supp Figure legends [file 41388_2020_1161_MOESM9_ESM.docx]

**Supplementary Figure Legends**

**Figure S1**. **Validation of deregulated target genes identified in the genome-wide analysis.**

(A) IPA comparative analysis identifying canonical pathways specifically and significantly enriched for H3K27me3 in GIC and NSC Bmi1^Over^. Numbers indicate –log(p-value), threshold for significance is 1.3. (B) Table showing overlap between unique concordant genes in the mGIC context and cell lines in the NIH Roadmap Epigenomics database (H3K27me3 datasets); significance is reported taking account of p-values adjusted for FDR (< 0.05). (C) Venn diagram showing the overlap between unique concordant genes in the mGIC context and a publicly available H3K27me3 hGIC dataset.

**Figure S2. Bmi1 represses the expression of EfnA5 but not of cell cycle inhibitors in mGIC.**

(A) Western blot and corresponding quantitive analysis showing increase EfnA5 expression in mGIC upon Bmi1 silencing as compared to control shScr (n=3). (B) Visualisation of the whole EFNA5 locus shows a differential H3K27me3 distribution (green) between hGIC (here named GSC as in Rheinbay 2013) and human NSC. (C) RNA microarray expression data for BMI1 and EFNA5 levels in hGIC and human NSC for the same cells shown in (B). (D) qPCR reveals no significant up-regulation of the expression the cell cycle inhibitors p16^Ink4A^, p19^Arf^ and p21^Cip1/Waf1^ in shBmi1 mGIC compared to the control (n=6). ***, p < 0.001; error bars represent ± SEM.

**Figure S3. Schematic representation of Proximity Ligation Assay (PLA).**

(A) Two primary antibodies (black) are used to recognise EfnA5 biotinylated probe (red) and H3K27me3 (green) at the *EfnA5* locus. Secondary antibodies (blue) enable detection of the PLA signal after ligation and amplification of conjugated oligonucleotide sequences (pink). (B) Model of the PcG-mediated regulation of EfnA5 locus. Schematic showing the proposed PcG-dependent mechanism regulating the expression of EfnA5 in GIC (upper panel) and NSC (lower panel) integrated within the existing knowledge of EfnA5 regulation in normal and neoplastic NSC.

**Figure S4. EfnA5 silencing rescues proliferation and migration in shBmi1 mGIC.**

(A) Western blot and corresponding quantitive analysis showing the levels of Bmi1 and EfnA5 in mGIC transduced with different combinations of shRNAs (n=2). (B) Representative pictures of the proliferation rate at different time points upon Bmi1 and/or EfnA5 silencing. The yellow mask represents the % of confluence (scale bar = 800µm). (C) Representative images of the gap closure assay. The yellow mask represents the % of confluence (scale bar = 800µm). (D) Representative pictures of neurosphere formation assay with corresponding quantification. The number of the neurospheres in shBmi1 condition is increased when EfnA5 expression is silenced (scale bar = 400 µm; n=3). *, p < 0.05; **, p < 0.01; ***, p < 0.001; ****, p < 0.0001; error bars represent ± SEM.

**Figure S5: EfnA5 mediates the reduced invasion of mGIC induced by Bmi1 silencing.**

(A) Representative micrographs of neurospheres with corresponding quantification from a neurosphere formation assay. The size of neurospheres is decreased in conditions with higher EfnA5 (n=3). (B) Quantitative analysis of invasion assay showing the percentage of invading nuclei at distances 0-120µm form the bottom of a collagen gel, showing that mGIC expressing high levels of EfnA5 partially lose their invasive potential. NSC represent the negative control (n=6). *, p < 0.05; **, p < 0.01; ***, p < 0.001; ****, p < 0.0001; error bars represent ± SEM.

**Figure S6. mGIC overexpressing Bmi1 show tumourigenic features.**

(A) Western blot and corresponding quantitive analysis showing the levels of Bmi1 and EfnA5 in mGIC transduced with different combinations of shRNAs (n=2). *, p < .05; **, p < .01, error bars represent ± SEM. (B) Histology of representative tumour areas arising from allografted shScr GIC with diffuse infiltration patter and strong positivity for GFP. Scale bar = 1 mm (top panel) and 250 μm (middle and bottom panel).

**Figure S7.** **EFNA5 mediates BMI1 function in hGIC.**

(A) Scatter plot with linear regression statistics showing the correlation between levels of BMI1 and EFNA5 for microarray data from TCGA (hthgu133a). (B) A schematic showing the method used for identifying cells with high and low expression of BMI1 and EFNA5 in the single-cell RNAseq datasets: red identifies cells with a BMI1^low^EFNA5^high^ profile and blue identifies cells with the inverse BMI1^high^EFNA5^low^ profile. (C) Representative images and quantification for EdU staining in U3033 cells after BMI1 knockdown and EFNA5 pathway inhibition. (D) Proliferation assays for U3033 after BMI1 knockdown with shRNA (upper) and concomitant BMI1 knockdown and EFNA5 pathway inhibition with recombinant proteins (lower). *, p < 0.05; **, p < 0.01; ***, p < 0.001; ****, p < 0.0001; error bars represent ± SEM. Scale bar =250 μm.

**Figure S8.** **Doxazosin effectively targets BMI1^high^/EFNA5^low^ hGIC *in vitro* and *in vivo*.**

(A) Doxazosin levels in the serum and brain homogenate of experimental mice over 24 hours after 50mg/kg subcutaneous injection (one mouse injected at each time-point). (B) Doxazosin levels in the serum and brain homogenate after concomitant administration of elacridar (Elac), Cnt+dose1 – vehicle control + 50mg/kg doxazosin, Cnt+dose2 – vehicle control + 100mg/kg doxazosin, Elac+dose1 – 100mg/kg + 50mg/kg doxazosin, Elac+dose2 – 100mg/kg elacridar + 100mg/kg doxazosin (n=2); *, p < 0.05; **, p < 0.01; ***, p < 0.001; ****, p < 0.0001; error bars represent SEM. (C) Treatment regime for in vivo co-administration of doxazosin (blue bars) and elacridar (red bars). Elacridar given orally four hours prior to subcutaneous doxazosin dose. (D) Histology of representative tumour areas with H&E and GFAP, OLIG2, SOX2 and human Vimentin; scale bars represent 2.5mm for human vimentin (low mag.), and 50µm for all others.
