# Supplementary material for: Polycomb-mediated repression of EphrinA5 promotes growth and invasion of glioblastoma
Source: Oncogene. 2020 Jan 27;39(12):2523–38. doi: 10.1038/s41388-020-1161-3 (PMC7082224; doi:10.1038/s41388-020-1161-3)
Supplement: Supplementary file 10 — M&M [file 41388_2020_1161_MOESM10_ESM.docx]

**Materials and Methods**

**Generation of mice and genotyping**

Transgenic *STOPFloxBmi1* mice were previously generated in our laboratory [^11^](#_ENREF_11). Activation of Bmi1 overexpression was obtained in embryos by crossing *STOPFloxBmi1* and *NestinCre* mice to generate double transgenic animals, as previously described [^11^](#_ENREF_11).

All procedures were carried out according to the Home Office Guidelines (Animals Scientific Procedures Act 1986, PPL 70/6452 and P78B6C064). Ear notches of transgenic mice and tails of transgenic embryos were digested in lysis buffer (50 mM Tris pH 8.0, 100 mM NaCl, 100 mM EDTA and 1% SDS) and Proteinase K (Biolabs) at 55°C, 700 rpm overnight, or for three hours respectively. The DNA was subsequently precipitated in isopropanol (Fisher Scientific) and dissolved in pre-warmed PCR-grade water. Genotypes of mice were determined by PCR as described previously [^11^](#_ENREF_11) [^7^](#_ENREF_7). Standard agarose gel electrophoresis was used to visualise the band of interest.

**Cultures**

Primary NSC cultures were prepared from E16.5 *STOPFloxBmi1;NestinCre* transgenic and control wild type embryos. Cerebral hemispheres were collected by brain dissection and NSC from the SVZ were isolated using a Papain dissociation kit (PDS, Worthington) and cultured as neurospheres (NS) or as adherent monolayer (adh). Primary mouse NSC were plated at 4x10^4^ mL^-1^ density and grown as NS in culture medium composed of DMEM/F12 (Invitrogen), mouse recombinant EGF (20ng/ml, Peprotech, ) and human recombinant b-FGF (20ng/ml, Peprotech, #AF-100-18B), 2% B27 (Invitrogen) and 1X Pen/Strep (Sigma Aldrich). Every 3-4 days the NS were mechanically dissociated and plated under the same conditions. For adh cultures, a single-cell suspension obtained from primary NS was plated at 10^5^ cm^-2^ density. NS were plated on to a 90 µg/mL matrigel-coated (Corning) flask or dish in culture medium.

Primary mouse *PTEN^F/F^;P53^F/F^* NSC and mGIC were obtained from Sebastian Brandner, Institute of Neurology, University College of London, UK. Deletion of *PTEN^F/F^;P53^F/F^* cassette in NSC was achieved by *in vitro* AdenoCre infection as described previously [^5^](#_ENREF_5). Primary mGIC were cultured as NS or adh monolayer in the same culture medium used for NSC. For adh mGIC, flasks or dishes were first double-coated with 0.01 mg/mL poly-L-lysine (Sigma Aldrich) in PBS for 30 minutes at room temperature, and then with 0.01 mg/mL Laminin (Sigma Aldrich) in PBS for 30 minutes at 37°C. Dissociation between passages was performed enzymatically with trypsin/EDTA (Invitrogen).

For EfnA5 pathway activation, mGIC were treated with 8µg/mL recombinant mouse EfnA5 Fc chimera (R&D Systems, #7396-EA) or IgG1 Isotype Control Clone 11711 (R&D Systems, #MAB002) pre-clustered with 4µg/mL anti-Fc (anti-mouse Alexa-546, ThermoFisher Scientific) in culture medium for 2 hours at 37°C. For prolonged assays the pre-clustered compound was refreshed every 24 hours.

To inhibit the activity of Ezh2, adh mGIC were plated at growth density with growth medium containing 5 µM Ezh2i (GSK-126, Chemietek) 24 hours prior to immunocytochemistry (ICC). Transient overexpression of JmjD3 was obtained by nucleofection (Amaxa, Lonza) of 10^6^ adh mGIC with 5 µg of FlagJmjD3LeGO-iC or control LeGO-iC (Addgene). 24 hours later, cells were fixed for ICC. For viral transduction, adh mGIC were plated at growth density in culture conditions 24 hours beforehand. Transduction was performed by adding 8 MOI of AdenoCre particles or 20 MOI of lentiviral particles containing the plasmids shBmi1 (Dharmacon, Clone Id V2LHS_48576, ), shEfnA5-32 (GeneCopoeia) or shEfnA5-34 (GeneCopoeia) overnight. shScramble and shmCherry plasmids, both containing a scramble sequence, were used as controls. Growth medium was then changed and the cells were allowed to recover before further assays were performed.

hGIC were obtained from HGCC (<http://www.hgcc.se>); they were cultured as adherent cells in Neurobasal and DMEM/F12 media (1:1 mix) containing N2 and B27 supplements (Invitrogen) and human recombinant FGF and EGF (10 ng/ml, PEPROTECH). Primaria dishes (BD Biosciences) coated with mouse laminin (Sigma-Aldrich) were used to allow adherent growth as described previously (Pollard et al., 2009). For EFNA5 pathway blockade, hGIC were treated with 4µg/mL recombinant human EFNA5 Fc chimera (R&D Systems, #374-EA-200), or EPHA4 Fc chimera (R&D Systems, #6827-A4-050) and EPHA5 FC chimera (R&D Systems, #541-A5-200), or IgG1 Isotype Control (R&D Systems, #110-HG-100), replaced daily in fresh media. For doxazosin treatment, cells were treated every other day with the stated concentration of doxazosin (Abcam, ab120754) in DMSO, with a final volume of 0.2% of complete medium. 0.2% DMSO was used as the vehicle control.

Cell lines used in this study are primary lines either derived from mouse brains or human tumours, they have been characterised by transcriptomic profiling and cultured according to best practice, including contamination-screening.

**ChIPSeq and RNASeq**

The chromatin immunoprecipitation (ChIP) assay was performed on two biological replicas of control NSC, two biological replicas of NSC Bmi1^Over^ (isolated from *NestinCre;STOPFloxBmi1*) and two mGIC [^5^](#_ENREF_5) according to previously published protocols with minor modifications [^2^](#_ENREF_2). Chromatin was sonicated to get fragments of 100 to 500 bp and immunoprecipitated with 10 µL of anti-H3K27me3 (Active Motif, #39155). ChIPSeq libraries were prepared following the Illumina protocol and ligated to standard PE adaptors and sequenced on a HiSeq2000. For RNASeq, 200 ng of total RNA was used to prepare RNASeq libraries using the TruSeq RNA kit from Illumina following the instructions provided in the supplier's manual, and sequenced on HiSeq2000. ChIPSeq and RNASeq data are available in the array express database (<http://www.ebi.ac.uk/arrayexpress/>), accession numbers will be available at time of publication.

RNASeq: Adaptor sequence and other artefacts were removed from sequence reads using the Trimgalore tool (www.bioinformatics.babraham.ac.uk/projects/trim_galore/). After trimming and quality control, reads were aligned to the mm10 mouse genome using STAR [^3^](#_ENREF_3). Further quality and biotype filtering was performed in R with NOISeq Bioconductor package [^10^](#_ENREF_10). Trimmed mean of M-values (TMM) normalization was applied to the dataset and differential expression (DE) analysis was performed using the Bioconductor package edgeR in R [^9^](#_ENREF_9), with a Quasi-Likelihood F-test (QLF) and an FDR cut-off of 0.05.

ChIPSeq: Adaptor sequence and other artefacts were removed from sequence reads using the Trimgalore tool. After trimming and quality control, reads were aligned to the mm10 mouse genome using Bowtie v2.3.4 (sourceforge.net/projects/bowtie-bio/files/bowtie2/), allowing up to one mismatch per read and discarding multi-mapped reads. Read totals after trimming and alignment varied between 14.8M and 3.5M in mGIC replicates, 6.4M and 4.5M in NSC Bmi1^Over^, 17.4M and 3.9M in NSC replicates.

After performing post-alignment quality checks, based on the number of final reads, quality scores and the analysis of fingerprint plots (not shown), we decided to discard the weakest samples and perform further ChIPSeq analysis only on one replicate per condition.

The MACS2 algorithm [^14^](#_ENREF_14) was used to call H3K27me3 peaks (subroutine *callpeak*) and perform the differential binding (DB) analysis (subroutine *bdgdiff*). The shifting model and the dynamic lambda were both disabled (as they are not recommended for histone modification analysis), the “broad mark” option was enabled and a minimum fold enrichment of 2 was selected with an FDR (--broad-cut-off) of 0.05. Finally, a minimum length of differential regions of 250 bp and a maximum gap (to merge nearby regions) of 200 bp were considered, in concomitance with a log likelihood ratio cut-off of 2 to call differential regions between conditions.

The Bioconductor package GenomicRanges [^6^](#_ENREF_6) was used to find overlaps of peak genomic coordinates between different conditions, i.e. mGIC vs NSC, or mGIC vs NSC Bmi1^Over^, whereas ChIPSeeker [^12^](#_ENREF_12) was used for the peak annotation and the promoter characterisation within 5 Kb.

RNASeq/ChIPSeq combined analysis: Because we would expect to see reduced expression of a gene harbouring an H3K27me3 peak in a promoter proximal region, we identified peaks in NSC, NSCBmi1^Over^ and mGIC datasets that showed significantly reduced gene expression at the locus containing an H3K27me3 peak and defined these peaks “expression concordant”. To characterize orthologous genes between murine and human datasets, biomaRt package in R was used [^4^](#_ENREF_4).

Hierarchical Clustering and Heatmaps: Clustering and heatmap generation for gene expression data and H3K27me3 peaks were performed using various packages in R such as gplots and RColorBrewer (<https://cran.r-project.org/>) and the Python suite deepTools v. 3.1.3 [^8^](#_ENREF_8).

Pathway analysis: We used Ingenuity Pathway Analysis (Qiagen, <https://www.qiagenbioinformatics.com/products/ingenuity-pathway-analysis/> ) to analyse pathways and networks that showed significant enrichment in the various gene sets generated by our analysis. The enrichment for each term was tested using hypergeometric distribution and p-values were corrected using an FDR procedure. All terms with a FDR <0.05 were considered enriched.

RMA log2 transformed signal intensity values were downloaded from the HGCC database website (hgcc.se). Pearson correlation coefficients and relative p-values were calculated using GraphPad Prism 7.

A schematic for the generation of the contingency tables in Fig.7A is shown in Fig.S7B. Ranges of expression for both BMI1 and EFNA5 were determined, separately for each single-cell RNASeq dataset analysed. The first quartile was chosen as the cut-off to define the “low expression” and “high expression” segments. Analysis with other cut-off values such as 15% and 50% (median) was also performed and the outcome did not show a significant difference (data not shown). Four regions of expression, each with a different BMI1/EFNA5 profile, can be obtained by combining the “low” and “high” segments for both genes. A contingency table with the counts for all regions was created for each dataset and both Fisher’s and Barnard’s exact tests were applied, to determine whether the distribution of counts among the aforementioned regions was non-random.

R v. 3.5.1 was used to run all aforementioned packages whereas Python v. 2.7.15 was used for MACS2 and deepTools routines.

**Western Blot**

Protein homogenates were obtained using RIPA buffer containing protease cocktail inhibitors (Santa Cruz Biotechnology). Protein concentration was determined by BCA assay (Pierce) and an equal amount (10-60µg) was loaded into a NuPAGE Novex 3-8% Tris-Acetate or a 4-12% Bis-Tris protein gel (ThermoFisher Scientific). Proteins were separated by SDS-PAGE and blotted onto a nitrocellulose membrane (Whatman). Membranes were blocked with 5% non-fat dried milk (Santa Cruz)-0.1% TWEEN-PBS and incubated with the primary antibody diluted in blocking solution according to each antibody’s recommendation: anti-Bmi1 (1:1000, mouse monoclonal Millipore, Clone F6, 05-637), anti-H3K27me3 (1:5000, rabbit polyclonal Millipore, 07-449), anti-EFNA5 (1:400, mouse monoclonal NOVUS Biologicals, Clone 1F12, H00001946-M01), anti-Tubulin (1:10000, mouse monoclonal Sigma Aldrich, T6199), anti-GAPDH (1:1000, mouse monoclonal Sigma, Clone 71.1, g8795), anti-H3 (1:50000 rabbit polyclonal Millipore, 07-690), anti-EphA2/A3/A4 (1:500, Abcam, ab196899), anti-Phospho-EphA2/A3/A4 (1:500, Abcam, ab62256), anti-ERK1/2 (1:1000, Cell Signalling Technology, 4695), anti-Phospho-ERK1/2 (thr202/Tyr204) (1:1000, Cell Signalling Technology, 4370). Membranes were then incubated with the peroxidase-conjugated mouse secondary antibody 1:5000 or rabbit secondary antibody 1:10000 (GE Healthcare, NA931V and NA934V respectively) and visualised on a film using ECL kit (GE Healthcare). Quantification of protein expression was performed by densitometric analysis and normalised with ImageJ software.

**Immunocytochemistry**

Adh mGIC were plated as a monolayer on coated coverslips. After fixation with 4% PFA (Sigma Aldrich) and permeabilisation with 0.1% TritonX-PBS (Sigma Aldrich), cells were blocked with 10% NDS (normal donkey serum, Millipore) / PBS and incubated overnight at 4°C with the primary antibodies: phalloidin (Phall-488 1:400, ThemoFisher Scientific, A12379), GFAP (1:500, rabbit polyclonal Dako Denmark, Z0334), Nestin (1:500, mouse monoclonal, Chemicon, MAB353). Coverslips were mounted with Vectashield mounting medium with DAPI (Vector Laboratories). The staining was developed using Alexa fluorescent conjugated secondary antibodies (ThemoFisher Scientific) and images were acquired using a Leica DM5000 Dual Camera Epifluoresence microscope. For phalloidin intensity measurements, the outline of positive cells was manually traced for ten randomly selected areas for each condition, and then staining intensity (integrated density) was measured for each cell with ImageJ software. Background was subtracted and resulting values were normalised against the control condition and expressed as a fold change. Statistical analysis (one-way ANOVA) was performed with GraphPad software. For cellular process length measurements, 10 random high power fields were captured for each condition and all cell processes (yellow arrowheads in Fig. 4A highlight example processes) were measured manually with ImageJ for each field. Measurements were taken in pixels then converted to µm using magnification properties of objectives used. Length values were then averaged for each condition, and are expressed as a fold change of the control condition. Statistical analysis (one-way ANOVA) was performed with GraphPad software.

For hGIC, shScramble or shBMI1 transduced cells were plated at growth density. After five days of daily treatment with 4ug/ml of recombinant mouse proteins (see above for details) cells were then fixed, blocked in 10% normal donkey serum (NSD, Millipore) and Tween20 (0.1%) in PBS. Primary antibody incubation with anti-Eph receptor A2/3/4 (Abcam, ab62256, 1:500) for 3 hours was followed by 1 hour incubation with AlexaFluor-546 (ThermoFisher). Coverslips were mounted with Vectashield mounting medium with DAPI (Vector Laboratories). Images were acquired using a Zeiss 710 confocal microscope and pictures were analysed with ImageJ software. Investigators were blinded to the group allocation during the analysis.

**EdU staining**

The Click‐iT® EdU Alexa Fluor® 594 Imaging Kit (ThermoFisher) was used to assess EdU incorporation following manufacturer’s instruction. Briefly, transduced hGIC were seeded on polylysine-coated coverslips in triplicates. The following day, cells began daily treatment with recombinant proteins (see above for details) for five days. On the fifth day, cells were treated with 10µM of EdU for three hours before being fixed in 4% paraformaldehyde (PFA), washed, blocked and incubated with Click‐iT® reaction cocktails containing Alexa Fluor® azide. Five representative images (40X magnification) of each sample were captured using a Zeiss 710 Confocal Microscope. The percentage of positive nuclei was calculated as the ratio between EdU‐positive cells and the total number of nuclei counted using ImageJ software.

**Cloning**

To overexpress JmjD3 in our cultures, the cassette FlagJmjD3 from the plasmid pCDNA-FlagJmjD3 (gift of Giuseppe Testa, IEO, Italy) was cloned into the lentiviral plasmid LeGO-iC (Addgene, #27362) for the production of lentiviral transducing particles (see below). Both plasmids were digested with BamHI-HF and NotI-HF restriction enzymes (New England Biolabs). The insert (FlagJmjD3) and the backbone were ligated at a 1:3 molar ratio with the T4 ligase enzyme (New England Biolabs) overnight at 16°C. Afterwards, TOP-10 bacteria one shot chemical competent cells (Invitrogen) were transformed with the ligation mixture according to the manufacturer’s instructions. Successful ligation was tested by BamHI-HF and NotI-HF restriction digestion of plasmid DNA (FlagJmjD3LeGO-iC) extracted from positive colonies and mutations were excluded by Sanger sequencing with four pairs of primers (F1 5’-CCTATTGACTGAGTCGCCCGGATC-3’, F2 5’-CAGCAGTAGCAGTAACAACAC-3’, F3 5’-AGTCCTGCATCAGGTGCTAC-3’, F4 5’-GCTAAATACGCACAGTACCAGG-3’, R1 5’-AATGGTCAGCGCCAGGAATG-3’, R2 5’-AGATGACGAGGAAACCGAGG-3’, R3 5’-TCACTCTCCCTCTCCTCCTG-3’, R4 5’-GAAGCCGCTTGGAATAAGGC-3’). Results were aligned to the mouse JmjD3 (KDM6B) sequence (genome assembly GRCm37) available on the NCBI BLAST website (<http://blast.ncbi.nlm.nih.gov/Blast.cgi>).

**Lentiviral production**

Second generation lentiviral particles were produced to deliver the following plasmids: FlagJmjD3LeGO-iC, shScramble, shBmi1, shmCherry, shEfnA5-32, shEfnA5-34, and Luciferase. 4-5x10^5^/cm^2^ HEK 293T cells were transfected with the lentiviral plasmids pCMV-G, pCMV-HIV1 and the plasmid of interest. After 48 hours in culture, the supernatant was collected, cell debris were removed by filtration (0.45 PVDF filter, Sartorius) and the lentiviral particles were precipitated with polyethylene glycol (Sigma Aldrich) and stored at at 4°C for 16 hours-4 days. Lentiviral particles were concentrated by centrifugation (30 minutes, 1500xg, 4°C), resuspended in sterile PBS and stored at -80°C.

Transducing units were then determined by plating 0.5 x10^5^ HEK 293T cells into 12-well-dishes and exposing them to serial dilutions, 10^-1^ to 10^-5^, of the lentiviral supernatant. Cells were collected 96 hours from the transduction and the percentage of GFP- or mCherry-positive cells was determined by FACS (BD FACS Canto II or Aria III Cell Sorter). The titre in transducing units per ml (TU/ml) was calculated according to the following formula: ((% positive cells/100) x no. transduced cells)/volume of virus (mL).

**RNA extraction, RT and qPCR**

For mGIC, 5x10^5^-10^6^ cells were harvested and RNA extraction was performed using Micro or Mini RNeasy kit (Qiagen). RNA was then treated with DNaseI (Invitrogen) and 1µg was retrotranscribed with SuperScriptIII (Invitrogen). 10-20ng of cDNA template and FAM labelled probes were used to perform Taqman assay (Invitrogen). Each reaction was performed in triplicate and normalisation was carried out against the housekeeping gene Gapdh. The following Taqman probes were used for gene expression analysis: **Gapdh** (Mm99999915_g1), **Bmi1** (Mm03053308_g1), **EfnA5** (Mm01237700_m1), **Jph3** (Mm00517489_m1), **Lrfn2** (Mm01281423_m1), **Scrt1** (Mm00459966_m1), **Cpne2** (Mm00467840_m1), **EphA2** (Mm00438726_m1), **Wnt7a** (Mm00437356_m1), **Cacna1g** (Mm00486572_m1), **Grin2a** (Mm00433802_m1), **Cacna1h** (Mm00445382_m1), **Cacna1a** (Mm00432190_m1), **Eya2** (Mm00802562_m1) **Cabp1** (Mm00600215_m1).

**Chromatin Immunoprecipitation followed by qPCR (qChIP)**

ChIP experiments were performed on mGIC cultured as NS. 5×10^6^ cells were dissociated into a single-cell suspension and resuspended in 10 mL of sterile PBS. Cells were cross-linked in 1% formaldehyde (Sigma Aldrich) for 15 minutes rocking at room temperature. Reaction was quenched with 0.125 M glycine (Santa Cruz Biotechnology) for 5 minutes rocking at room temperature. The mixture was centrifuged for 5 minutes at 4°C, 2000 rpm and the pellet was washed twice with cold sterile PBS to remove any formaldehyde residue. The cell pellet was then incubated with Washing Buffer (10mM HEPES/KOH pH 7.9, 85mM KCl, 1mM EDTA, 0.5% IGEPAL, protease inhibitor cocktail) for 30 min on ice. After 5 minutes centrifugation at 4°C, 4000 rpm, the pellet containing the nuclei was incubated in Lysis Buffer (1 mM PMSF, 50 mM Tris/HCl pH 7.4, 1% SDS, 0.5% Empigen BB, 10 mM EDTA, 1X protease inhibitor cocktail Roche) on ice for 30 minutes. Chromatin was then transferred into a Bioruptor Pico Microtube (Diagenode) and sonicated with a Bioruptor machine (Diagenode) for 7 cycles, 30 seconds ON/ 30 seconds OFF to achieve an average fragment size of 100 – 1,000 base pairs (bp). Beads (Invitrogen) were blocked with 2.5 mg/mL BSA (Sigma Aldrich) and 1 mg/mL salmon sperm (Sigma Aldrich) for 2 hours rocking at 4°C**.** Afterwards, the chromatin was precleared for 2 hours at 4°C with 1 mg of blocked beads (Invitrogen). 15-20 µg of precleared chromatin were immunoprecipitated with 2.5 µg of rabbit polyclonal H3 (Abcam, #A3912) or rabbit polyclonal H3K27me3 (Millipore, #07-449) antibodies overnight rocking at 4°C. The immunocomplexes were then incubated for 3-5 hours with rocking at 4°C and with 1 mg of beads previously blocked. Bead-immunocomplexes were washed for 5 minutes, rocking at 4°C with the following buffers: Washing Buffer A (50 mM Tris pH8.0, 150 mM NaCl, 1 mM EDTA, 0.1% SDS, 1% IGEPAL, 0.5% deoxycholate, protease inhibitor cocktail), Washing Buffer B (50 mM Tris pH8.0, 500 mM NaCl, 1 mM EDTA, 0.1% SDS, 1% IGEPAL, 0.5% deoxycholate, protease inhibitor cocktail), Washer Buffer C (50 mM Tris pH8.0, 250 mM LiCl, 1 mM EDTA, 1% IGEPAL, 0.5% deoxycholate, protease inhibitor cocktail). Immunocomplexes were eluted from the beads with an incubation for 40 minutes at 65°C, 1400 rpm with Elution Buffer (1% SDS, 0.1 M NaHCO_3_) Proteins were reverse-crosslinked for 15 minutes at 37°C 650 rpm with RNaseA and degraded with Proteinase K overnight at 65°C, 650 rpm. DNA was extracted with standard phenol:chloroform:isamyl alcohol (25:24:1) precipitation and air dried. 2 µL of resuspended DNA was used to perform SYBR Green qPCR (Applied Biosystems) and determine the abundance of specific sequences within the immunocomplexes. Primers used for the EfnA5 locus: **PS3.2 Fwd** 5’-AAAGATTGCTTTTCCTTCTGAG-3’, **PS3.2 Rev** 5’-CTATCCTAGGCTAAGACACTGT-3’, **PS3.5 Fwd** 5’-ATTGCACAAGAATACTCCTTAT-3’, **PS3.5 Rev** 5’-TGACTCACTTGTACGGGAACAA-3’. H3-immunoprecipitated chromatin was used as reference for normalisation and enrichments were calculated using the ChIP‐qPCR Data Analysis ΔΔCt method. Data are shown as fold changes of the percentage of INPUT and results of at least two independent biological replicates.

**Proximity Ligation Assay, including probe generation and biotinylation**

We cloned a 578 bp region spanning chr17:62687621-62688198 for EfnA5 locus into a TOPO TA Cloning vector (Thermo Fisher Scientific, 450641) according to manufacturer's instructions. FW: ACTGGTGGGACTCTCTCAAATTT and REV: AATGACTCACTTGTACGGGAACAA were used to clone the EfnA5 locus. NEB 5-alpha Competent E. coli cells (New England Biolabs, C2987H) were used for bacterial transformation and extraction of vector DNA. 1µg of plasmid DNA was then used as template to generate a biotintylated probe using Nick Translation Kit (Roche, 10976776001) and Biotin-14-dATP (Thermo Fisher Scientific, 19524-016). Plasmid DNA was incubated with nick translation reaction mix for 2 hours at 16 °C and stored at - 20 °C after the addition of 25 µM EDTA pH 8.0.

Cells were seeded on glass coverslips at a density of 10^5^/cm^2^. Cells were then incubated in PBS 1X / Fish Skin Gelatin (FSG, Sigma G7765) supplemented with normal donkey serum at 10% and Triton-X at 0.1% for 1h RT in a humidity chamber. Slides were then dehydrated with washes of increasing ethanol percentages, 50%, 70%, 90% and 100% at RT, 10 min each. Dehydration was followed by an incubation in 1mM EDTA pH 8.0 at 40-45 °C for 20 min. Slides were incubated at 37 °C for 15 min. in 4-4.5 pH pepsin solution prepared as following: 100 mg of Pepsin (Sigma P7000), 5 mL of 1M TrisHCL pH 8.0, 2 mL of 200mM CaCl2, 2 mL of 0.5 M EDTA pH 8.0, 200µl of 5 M NaCl in a final volume of 100 mL of distilled water. Cells were then washed in PBS 1X for 5 min.

The probe was prepared in a final volume of 20 µL/slide using 2% of biotinylated probe, 1% mouse Cot-1 DNA (Thermo Fisher Scientific, 18440-016) and 70% of Hybridization Buffer: 500 µL of Formamide , 100 µL of Dextran Sulfate 50%, 100 µL of SSC 20X and 300 µL of distilled water. Probe mixture was denatured at 80 °C for 10 min. and immediately transferred to slides. Slides and probe mixture were heated once again at 80 °C for 10 min to encourage probe annealing, and then incubated overnight at 37 °C in a humidity chamber. Following overnight incubation, slides were washed 3 times in SSC 2X and 0.1% NP-40 for 15 min. and then in PBS 1X for 5 min. At this point Duolink® In Situ Far Red Kit Goat/Rabbit (Sigma, DUO92013, DUO92003, DUO92005) was used. Slides were blocked using the provided solution at RT for 45 min. and then incubated at 4 °C overnight with polyclonal rabbit anti-H3K27me3 (Millipore, 07-449) diluted 1:200 and goat anti-biotin (Vector Laboratories, SP3000) 1:200 in antibody dilution solution. The next day cells were washed 3 times in wash buffer A for 5 min. and then incubated with PLA probes PLUS and MINUS diluted 1:5 in antibody dilution solution at 37 °C for 1 hour in humidity chamber. After 3 washes in wash buffer A for 5 min. ligation was performed using ligase diluted 1:40 in ligation stock 1:5 in water at 37°C for 30 min. in humidity chamber. Slides were washed 3 times in wash buffer A for 5 min. Amplification was performed using polymerase diluted 1:80 in amplification stock 1:5 in water at 37°C for 100 min in humidity chamber. Slides were washed 3 times in wash buffer B for 10 min and then wash buffer 0.01X for 5 min. Anti-mCherry antibody (Abcam, clone 1C51, ab125096) was diluted in 0.1% BSA , 1% normal donkey serum and PBS 1X 1:250 and incubated overnight at 4°C to detect transfected cells. Slides were mounted using Vectashield mounting medium with DAPI (Vector Laboratories).

***In Vitro* Functional Assays**

*Proliferation Assay*

1x10^3^ cells of adh mGIC were seeded in each well of a 96-well plate. Using an IncuCyte ZOOM Live-Cell Analysis System (EssenBioScience), wells were scanned and imaged every 2 hours for 2 days at 4X magnification. After background and cell debris subtraction, proliferation rate was assessed with the IncuCyte ZOOM Software (EssenBioScience) as occupied area (% confluence). At least two biological replicas with three technical replicates each are shown for every experiment.

1x10^4^ cells of hGIC were seeded in each well of a 24-well plate. Cell counting was performed with Tryptan blue staining using a haemocytometer. At least two biological replicas with three technical replicates each are shown for every experiment.

*Wound Healing Scratch Assay*

5x10^4^ cells of adh mGIC were seeded per well in a 96-well plate. A scratch was then made with a WoundMaker™ 96-pin tool (EssenBioScience) and 50 µg/mL mitomycin C (Sigma Aldrich) was added to the growth medium to inhibit the cell proliferation. Images were taken every 2 hours for 16 hours at 4X magnification with IncuCyte ZOOM Live-Cell Analysis System (EssenBioScience). After background and cell debris subtraction, wound closure was determined with the IncuCyte ZOOM Software (EssenBioScience). At least two biological replicas with three technical replicas each were shown for every experiment.

*Invasion Assay*

5x10^3^ E16.5 WT NSPC or mGIC cells were resuspended with 100 µL of the collagen gel mixture (4 mg/mL of Rat Tail Collagen Type I High Concentration (Corning) and 10% MEM (Gibco)) and plated in a 96-well plate. The mixture was spun for 10 minutes at 4°C, 1000 rpm. After 30 minutes incubation at 37°C, to allow the polymerisation of the collagen, growth medium was added on top of the gel in each well. 96 hours later, cells were then fixed with 4% PFA-PBS for 30 minutes at room temperature and stained with 1 ng/mL Hoechst (Sigma Aldrich) in 1X TNE buffer (Sigma Aldrich) for one hour at room temperature. Images were taken with the INCell 2200 (GE Healthcare) and analysis of the invading nuclei was performed with Developer Toolbox software (GE Healthcare) after background subtraction. Two biological replicas with at least two technical replicas each are shown for every experiment.

*Neurosphere Formation Assay*

2.5x10^2^ mGIC were plated in each well of a 96-well plate and pictures were acquired using a Nikon Eclipse TE2000-S microscope after 96 hours in culture conditions. Neurosphere area and number were determined with ImageJ software. Two biological replicas with six technical replicas each are shown for every experiment.

**Orthotopic transplantation of GIC into NODSCID mice and bioluminescence imaging (BLI)**

All the *in vivo* procedures were carried out under Home Office approval (Animals Scientific Procedures Act 1986, PPL 70/7275 and P78B6C064). 6-12 week old NODSCID mice were anaesthetised with 6.4 µL per gram of body weight of the anaesthetic mixture (1mL of Narketan-10 (Vetoquinol), 0.5mL Rompun (Bayer) and 5.7 mL distilled water) by intraperitoneal injection. The scalp of each mouse was incised with a scalpel and the skull was exposed. 10 µl of sterile PBS containing 5x10^5^ mGIC were slowly injected with a 26 gauge Hamilton syringe needle into the right cerebral hemisphere with the following coordinates from the bregma suture: 2 mm posterior, 2 mm lateral, 4 mm deep, 10° angle. The scalp was then sutured with 4-0 Coated Vicryl Suture (Ethicon) and the mice were allowed to recover on a heat-mat. Post-operative checks were performed twice a day for five days after surgery and every day thereafter. Mice were culled when symptomatic unless otherwise stated. Tumour formation and growth was assessed by BLI for mGIC xenografts. Mice were scanned every seven days from day 26 after transplantation, using IVIS Lumina III imaging system (PerkinElmer). Mice were intraperitoneally injected with 10 µL per gram of body weight of 15 mg/mL D-Luciferin D-Luciferin (Melford Laboratories) in DPBS (Gibco) and immediately anaesthetised with isoflurane (Abbott Laboratories). Images were acquired between 10 and 25 minutes after D-Luciferin injection, luminescence was recorded with a series of three minutes scans with large binning. Total flux (p/s) was measured with Living Image 4.3.1 software. To detect a reduction of an endpoint (Cohen’s d [effect size] =2) at a statistical significance level (alpha level) of p<0.001 we estimated that 10 mice were needed per group, including mice added to compensate for anticipated losses (premature death, no graft growth, other losses [^1^](#_ENREF_1).

**Doxazosin level measurements in mice**

Drug suspension: Each 50mg of doxazosin or elacridar (Insight Biotechnology) were dissolved in warmed DMSO, the resultant suspension was then vortexed with Kolliphor HS15 after heating to 50⁰C then final injection mixture made up with warm PBS.

For experiments to determine doxazosin levels, experimental mice were subcutaneously injected with doxazosin at 50 or 100 mg/kg in 5% DMSO / 10% Kolliphor / PBS. Where concomitant doxazosin and elacridar was used, elacridar in 10% DMSO / 20% Kolliphor / PBS was administered by oral gavage four hours before doxazosin dose. 5% DMSO / 10% Kolliphor HS15 / PBS was used as a vehicle control for doxazosin and was used for a vehicle control for elacridar.

At stated time-points mice were culled with pentobarbital terminal anaesthesia and samples removed for doxazosin level assessment using published methods [^13^](#_ENREF_13). Blood was extracted by cardiac puncture and brains were removed. Blood was allowed to clot, centrifuged at 4,000 rpm for 10 min., and serum taken for analysis. Forebrain was dissected and weighed, then homogenised in PBS using a Qiagen TissueRuptor (9002757). After centrifugation, homogenate supernatant was then mixed 1:1 with chloroform, centrifuged at 15,000 rpm for 5 min. and the aqueous (top) portion was taken for drug measurement. Doxazosin levels were then analysed using liquid chromatography – mass spectrometry at the QMUL Mass Spectrometry Laboratory, Mile End, UK.

***In vivo* treatment with doxazosin**

Tumour naïve mice treated once daily for 21 days with 50mg/kg, and 12 days with 100mg/kg did not suffer any weight loss, or show any behavioural changes. For *in vivo* doxazosin treatment of tumours, 100mg/kg of doxazosin was given daily by subcutaneous injection and 100mg/kg elacridar was given by oral gavage every second or third day, 4 hours prior to doxazosin dose (for schematic see figure S8D). Mice were assigned to treatment groups keeping number of male and females in each group equal. Experimental animals (n=8 for each group) were treated with elacridar and vehicle control, or elacridar and doxazosin without blinding. Mice were culled after treatment and brains removed for histological assessment.

**Immunohistochemistry quantification**

Tumour area: Human vimentin immunohistochemistry slides were scanned on a slide scanner, then an analysis protocol was composed on Definiens software for automated area quantification. Proliferation: High powered fields were randomly captured in tumour areas (four per condition), and Ki67 positive cells were counted with ImageJ software. Investigators were blinded to the group allocation during the analysis.

**Statistical analysis**

Statistical analysis was performed using GraphPad software unless otherwise stated. Significance was determined with t-test, one-way ANOVA (with Sidak’s test) or two-way ANOVA as appropriate, unless otherwise stated, and displayed as the mean ± standard error (SEM). p < 0.05 was considered significant. Significance was indicated with asterisks: *, p < 0.05; **, p < 0.01; ***, p < 0.001; ****, p < 0.0001. At least three technical and two independent biological replicates were used in each experiments. All variables were assumed to be normally distributed unless otherwise stated. Outliers were considered those data points furthest from the median value.

**References**

1 Acquati S, Greco A, Licastro D, Bhagat H, Ceric D, Rossini Z *et al*. Epigenetic regulation of survivin by bmi1 is cell type specific during corticogenesis and in gliomas. Stem cells (Dayton, Ohio) 2013; 31: 190-202.

2 Cuddapah S, Jothi R, Schones DE, Roh TY, Cui K, Zhao K. Global analysis of the insulator binding protein CTCF in chromatin barrier regions reveals demarcation of active and repressive domains. Genome Res 2009; 19: 24-32.

3 Dobin A, Davis CA, Schlesinger F, Drenkow J, Zaleski C, Jha S *et al*. STAR: ultrafast universal RNA-seq aligner. Bioinformatics (Oxford, England) 2013; 29: 15-21.

4 Durinck S, Spellman PT, Birney E, Huber W. Mapping identifiers for the integration of genomic datasets with the R/Bioconductor package biomaRt. Nat Protoc 2009; 4: 1184-1191.

5 Jacques TS, Swales A, Brzozowski MJ, Henriquez NV, Linehan JM, Mirzadeh Z *et al*. Combinations of genetic mutations in the adult neural stem cell compartment determine brain tumour phenotypes. The EMBO journal 2010; 29: 222-235.

6 Lawrence M, Huber W, Pages H, Aboyoun P, Carlson M, Gentleman R *et al*. Software for computing and annotating genomic ranges. PLoS Comput Biol 2013; 9: e1003118.

7 Marino S, Vooijs M, van Der Gulden H, Jonkers J, Berns A. Induction of medulloblastomas in p53-null mutant mice by somatic inactivation of Rb in the external granular layer cells of the cerebellum. Genes & development 2000; 14: 994-1004.

8 Ramirez F, Ryan DP, Gruning B, Bhardwaj V, Kilpert F, Richter AS *et al*. deepTools2: a next generation web server for deep-sequencing data analysis. Nucleic Acids Res 2016; 44: W160-165.

9 Robinson MD, McCarthy DJ, Smyth GK. edgeR: a Bioconductor package for differential expression analysis of digital gene expression data. Bioinformatics (Oxford, England) 2010; 26: 139-140.

10 Tarazona S, Furio-Tari P, Turra D, Pietro AD, Nueda MJ, Ferrer A *et al*. Data quality aware analysis of differential expression in RNA-seq with NOISeq R/Bioc package. Nucleic Acids Res 2015; 43: e140.

11 Yadirgi G, Leinster V, Acquati S, Bhagat H, Shakhova O, Marino S. Conditional activation of Bmi1 expression regulates self-renewal, apoptosis, and differentiation of neural stem/progenitor cells in vitro and in vivo. Stem cells (Dayton, Ohio) 2011; 29: 700-712.

12 Yu G, Wang LG, He QY. ChIPseeker: an R/Bioconductor package for ChIP peak annotation, comparison and visualization. Bioinformatics (Oxford, England) 2015; 31: 2382-2383.

13 Zhang P, de Gooijer MC, Buil LC, Beijnen JH, Li G, van Tellingen O. ABCB1 and ABCG2 restrict the brain penetration of a panel of novel EZH2-Inhibitors. Int J Cancer 2015; 137: 2007-2018.

14 Zhang Y, Liu T, Meyer CA, Eeckhoute J, Johnson DS, Bernstein BE *et al*. Model-based analysis of ChIP-Seq (MACS). Genome Biol 2008; 9: R137.
